# Tissue-Resident-Memory CD8^+^ T Cells Bridge Innate Immune Responses in Neighboring Epithelial Cells to Control Human Genital Herpes

**DOI:** 10.3389/fimmu.2021.735643

**Published:** 2021-09-06

**Authors:** Tao Peng, Khamsone Phasouk, Catherine N. Sodroski, Sijie Sun, Yon Hwangbo, Erik D. Layton, Lei Jin, Alexis Klock, Kurt Diem, Amalia S. Magaret, Lichen Jing, Kerry Laing, Alvason Li, Meei-Li Huang, Max Mertens, Christine Johnston, Keith R. Jerome, David M. Koelle, Anna Wald, David M. Knipe, Lawrence Corey, Jia Zhu

**Affiliations:** ^1^Department of Laboratory Medicine and Pathology, University of Washington School of Medicine, Seattle, WA, United States; ^2^Vaccine and Infectious Disease Division, Fred Hutchinson Cancer Research Center, Seattle, WA, United States; ^3^Department of Microbiology and Virology Program, Blavatnik Institute, Harvard Medical School, Boston, MA, United States; ^4^Department of Biostatistics, University of Washington, Seattle, WA, United States; ^5^Department of Medicine, University of Washington School of Medicine, Seattle, WA, United States; ^6^Department of Global Health, University of Washington School of Medicine, Seattle, WA, United States; ^7^Benaroya Research Institute, Seattle, WA, United States; ^8^Department of Epidemiology, University of Washington, Seattle, WA, United States; ^9^Institute for Stem Cell & Regenerative Medicine, University of Washington, Seattle, WA, United States

**Keywords:** tissue-resident-memory T cells (TRM), innate antiviral response, cell-intrinsic immunity, IFI16 restriction factor, tissue microenvironment, human genital herpes

## Abstract

Tissue-resident-memory T cells (TRM) populate the body’s barrier surfaces, functioning as frontline responders against reencountered pathogens. Understanding of the mechanisms by which CD8TRM achieve effective immune protection remains incomplete in a naturally recurring human disease. Using laser capture microdissection and transcriptional profiling, we investigate the impact of CD8TRM on the tissue microenvironment in skin biopsies sequentially obtained from a clinical cohort of diverse disease expression during herpes simplex virus 2 (HSV-2) reactivation. Epithelial cells neighboring CD8TRM display elevated and widespread innate and cell-intrinsic antiviral signature expression, largely related to IFNG expression. Detailed evaluation *via* T-cell receptor reconstruction confirms that CD8TRM recognize viral-infected cells at the specific HSV-2 peptide/HLA level. The hierarchical pattern of core IFN-*γ* signature expression is well-conserved in normal human skin across various anatomic sites, while elevation of IFI16, TRIM 22, IFITM2, IFITM3, MX1, MX2, STAT1, IRF7, ISG15, IFI44, CXCL10 and CCL5 expression is associated with HSV-2-affected asymptomatic tissue. In primary human cells, IFN-*γ* pretreatment reduces gene transcription at the immediate-early stage of virus lifecycle, enhances IFI16 restriction of wild-type HSV-2 replication and renders favorable kinetics for host protection. Thus, the adaptive immune response through antigen-specific recognition instructs innate and cell-intrinsic antiviral machinery to control herpes reactivation, a reversal of the canonical thinking of innate activating adaptive immunity in primary infection. Communication from CD8TRM to surrounding epithelial cells to activate broad innate resistance might be critical in restraining various viral diseases.

## Introduction

During primary infection by a microbe, the host innate immune mechanisms provide the initial recognition of pathogen-associated molecular patterns and trigger off signaling pathways that help to control the pathogen and activate adaptive immune mechanisms (1). The adaptive immune mechanisms then provide a lasting antigen-specific immunity that controls the pathogen upon re-infection or recurrent infection. Recent evidence indicates that tissue-resident-memory T cells (TRM) not only infiltrate the infection site but also persist in tissue for prolonged time periods, offering local protection in a timely and effective manner against pathogens reencountered at barrier surfaces (2–11). Murine studies of various viral infection models reveal that CD8TRM serve as sentinels for sensing, alarming and recruiting both innate and adaptive immune cells, thus providing tissue-wide effective containment, superior to their blood counterparts (5–7, 12). However, our understanding of the mechanisms underlying local immune protection against re-infection is less clear in a natural human disease condition.

Human herpes simplex virus (HSV) infection is a recurrent chronic disease, where latency and reactivation could occur over a large anatomic space involving the peripheral nervous system and the body’s barrier surface, including skin and oral/genital mucosa (13). Enhanced peripheral nerve innervation has been observed during intermittent HSV recurrence (14, 15). The dense neurite network likely serves as an evasion strategy for escaping host immune surveillance at the local site of virus reactivation (16). Nevertheless, clinical and virologic studies using frequent swabbing and sensitive PCR detection have shown that HSV reactivation in human is common, mostly asymptomatic and in short duration (17–19), indicating successful control of reactivated virus before overt herpetic lesion formation. The mechanistic insights of how human hosts coordinate such rapid and widespread protection are yet to be elucidated.

Detailed characterization of the spatiotemporal dynamics and functional phenotypes of the immunological milieu at the local site of recurrence have shown that CD8^+^ TRM (CD8TRM) cells, including HSV-2 specific CD8TRM, persist at prior lesion sites long after healing and accumulate at the dermal-epidermal junction (DEJ) near sensory nerve endings, where the reactivating viruses are released (10, 20). The persisting DEJ CD8TRM cells express signature genes for T cell activation and cytotoxic effector function, and genes encoding antiviral cytokines and chemokines (21). Potent cytotoxic activity and high CD8TRM effector-to-target ratios have been associated with rapid clearance and early containment of pathogens (11). It is largely unclear how a limited number of CD8TRM can provide tissue-wide protection and what the role cytokines play in a non-cytolytic fashion.

Here, we report that epithelial cells neighboring CD8TRM display an enhanced antiviral defense mechanism. Through investigation of 135 sequential skin biopsies from a cohort of 42 study participants, who represent a wide spectrum of clinical outcomes, our studies indicate that CD8TRM activate the innate and cell-intrinsic immunity in surrounding epithelial cells to provide tissue-wide resistance and restriction, directed at limiting pathogen spread and subsequent disease.

## Materials and Methods

### Study Participants and Biopsy Collection

Healthy, HSV-2 seropositive adults were recruited at the University of Washington Virology Research Clinic in Seattle, WA. HSV-2 serostatus was determined by Western blot as previously described (22). All participants were HIV seronegative and biopsy procedures were conducted as described previously (10, 23). The study protocol was approved by the University of Washington Human Subjects Review Committee and all participants provided written informed consent. Three millimeter diameter punch biopsies were obtained during clinical recurrences from active lesion sites and at the same sites post-healing, as previously described (10, 11, 21). Biopsies taken from acute lesions included half of the biopsy covering the vesicle area and the other half covering the immediately adjacent erythematous skin area. Biopsies from the post-healing time period were obtained from the predominant lesion area, usually contiguous to the prior biopsy site. Control skin biopsies were taken from normal epithelialized inner arm skin and/or the contralateral anatomic site of HSV reactivation (**Table 1**).

**Table 1:** Demographic information of study participants and the types of biopsy tissue studied.

Participants	HSV Status	Gender	Age	Years with HSV	Lesion site	Sequential biopsy tissue	Notes
1	HSV2	F	42.5	9.4	Perineal	Lesion, healed, Control	LCM keratinocytes
2	HSV1 & 2	F	62.3	32.9	Buttock	Lesion, 8wph, Control	LCM keratinocytes
3	HSV2	F	48.5	0.6	Buttock	Lesion, 4wph, 8wph, Control	LCM keratinocytes
4	HSV1 & 2	F	55	21.8	Buttock	8wph, Control	LCM keratinocytes
5	HSV1 & 2	F	56	34.7	Buttock	8wph, Control	LCM DEJCD8, Langerhans cell
6	HSV1 & 2	F	63.2	26.3	Buttock	8wph, Control	LCM DEJCD8, Langerhans cell
7	HSV1 & 2	F	48.1	30.6	Vulva	8wph, Control	LCM DEJCD8, Langerhans cell
8	HSV2	F	51.4	32.2	Buttock	8wph, Control	LCM DEJCD8, Langerhans cell
9	HSV1 & 2	F	42.9	23.7	Buttock	8wph, Control	LCM DEJCD8, Langerhans cell
10	HSV2	M	53.1	30.1	Buttock	8wph, Control	LCM DEJCD8, Langerhans cell
11	HSV2	F	33.3	1.6	Buttock	8wph, Control	LCM DEJCD8, Langerhans cell
12	HSV2	F	43	22	Buttock	8wph, Control	LCM DEJCD8, Langerhans cell
13	HSV2	F	37.9	19.2	Vulva	Lesion, 8wph, Control	Tissue Transcriptional profiling
14	HSV1 & 2	M	56.9	17.2	Buttock	Lesion, 8wph, Control	Tissue Transcriptional profiling
15	HSV2	F	57.8	34.2	Buttock	Lesion, 8wph, Control	Tissue Transcriptional profiling
16	HSV2	F	35.3	8.4	Glut Cleft	Lesion, 8wph, Control	Tissue Transcriptional profiling
17	HSV1 & 2	F	66.6	37.2	Buttock	Lesion, 8wph, Control	Tissue Transcriptional profiling
18	HSV1 & 2	F	48.1	26.9	Perineum	Lesion, 8wph, Control	Tissue Transcriptional profiling
19	HSV1 & 2	F	39.5	1.7	Labia	Lesion, 8wph, Control	Tissue Transcriptional profiling
20	HSV2	F	61.5	19	Buttock	Lesion, 8wph, Control	Tissue Transcriptional profiling
21	HSV2	F	62.4	40.9	Buttock	Lesion, 8wph, Control	Tissue Transcriptional profiling
22	HSV1 & 2	F	34.5	4.6	Vulva	Lesion, 8wph, Control	Tissue Transcriptional profiling
23	HSV2	F	60.6	30.8	Perineum	Lesion, 8wph, Control	Tissue Transcriptional profiling
24	HSV1 & 2	F	45	4.1	Buttock	Lesion, 8wph, Control	Tissue Transcriptional profiling
25	HSV1 & 2	F	48.9	44.2	Buttock	2wph, 8wph, Control	Tissue Transcriptional profiling
26	HSV2	F	53.4	29.6	Perineal	Lesion, 2wph, 8wph	Tissue Transcriptional profiling
27	HSV1 & 2	F	69.5	50.3	Buttock	Lesion, 2wph, 8wph	Tissue Transcriptional profiling
28	HSV1 & 2	M	33.8	4.9	Buttock	Lesion, 2wph, 8wph, Control	Tissue Transcriptional profiling
29	HSV1 & 2	F	46	11.8	Perianal	Lesion, 2wph, 8wph, Control	Tissue Transcriptional profiling
30	HSV2	F	54.9	36.9	Vulva	Lesion, 2wph, 8wph, Control	Tissue Transcriptional profiling
31	HSV1 & 2	F	53.5	34.9	Labia	Lesion, 2wph, 8wph, Control	Tissue Transcriptional profiling
32	HSV2	M	66.8	41.9	Buttock	Lesion, 2wph, 8wph, Control	Tissue Transcriptional profiling
33	HSV1 & 2	F	37.4	16.9	Thigh	Lesion, 2wph, 8wph, Control	Tissue Transcriptional profiling
34	HSV2	F	65.7	41.3	Vulva	Lesion, 2wph, 8wph, Control	Tissue Transcriptional profiling
35	HSV2	M	52.8	24	Buttock	Lesion, 2wph, 8wph, Control	Tissue Transcriptional profiling
36	HSV1 & 2	F	44.7	13.3	Mons	Lesion, 2wph, 8wph, Control	Tissue Transcriptional profiling
37	HSV1 & 2	F	27.6	3.4	Mons	Lesion, 2wph, 8wph, Control	Tissue Transcriptional profiling
38	HSV1 & 2	F	39.7	11.2	Perineal	Lesion, 2wph, 8wph, Control	Tissue Transcriptional profiling
39	HSV1 & 2	F	50.6	18	Buttock	Lesion, 2wph, 8wph, Control	Tissue Transcriptional profiling
40	HSV2	F	65.5	40.3	Buttock	Lesion, 2wph, 8wph, Control	Tissue Transcriptional profiling
41	HSV1 & 2	F	23.9	1.1	Labia	Lesion, 2wph, 4wph, 8wph, Control	In situ characterization only
42	HSV2	F	71.6	46.8	Buttock	Lesion, 2wph, 4wph, 8wph, Control	In situ characterization only

### HSV Detection in Biopsy Tissue

All tissue samples were fresh frozen in optimal cutting temperature (OCT) compound and stored at -80°C until processing. We performed immunofluorescence staining using rabbit polyclonal antibody to detect HSV-2 antigen (Agilent, Cat# B0116, RRID: AB_2335703) in skin lesion biopsies. We extracted DNA from six 8-micron thick tissue sections for each biopsy and used a sensitive PCR assay to detect HSV-2 DNA in lesion and post healed biopsies for virus quantification and screening for subclinical shedding (24).

### Viral Stocks and Cell Culture

Viral stocks utilized in this study include HSV-1 wildtype strain KOS and recombinant strain K26 which contains VP26-GFP fusion gene (a generous gift from Dr. Prashant Desai, Johns Hopkins University, Baltimore, MD), and HSV-2 wildtype strains HG52 and 186. Viral titers were determined by titration in Vero cells. Primary human adult keratinocytes were purchased from Lifeline Cell Technology (#FC-0025). Cells were cultured in DermaLife^®^ Basal Medium with DermaLife K LifeFactors (Lifeline Cell Technology Cat # LL-0007) as recommended by the manufacturer. Human diploid fibroblasts were cultures from skin biopsies as previously described (25), and used at low passage. CRISPR-Cas9 IFI16 knockout and Cas9 control cell lines were generated in human foreskin fibroblasts (Hs27 cell line; ATCC, CRL-1634, RRID: CVCL_0335) as described previously (26), using the IFI16 gRNA sequence: GUUCCGAGGUGAUGCUGGUU and maintained under puromycin selection (1 µg/mL) in Dulbecco’s modified Eagle’s medium (DMEM) supplemented with 10% (vol/vol) fetal bovine serum (FBS) and penicillin-streptomycin.

### Laser Capture Microdissection of Keratinocytes From Genital Skin Biopsies

We utilized a rapid immunofluorescence staining method (<15 minutes) to identify CD8^+^ T cells located near the dermal-epidermal junction from skin biopsies (11, 21). We then used the Zeiss PALM Microbeam system to perform laser capture microdissection of selected individual basal keratinocytes in the vicinity of CD8 cells and catapult them to designated tubes in a completely automated process. On average, 100 cells were captured per skin biopsy and total RNA was isolated and processed for gene expression analysis *via* the Illumina array platform.

### RNA Extraction, Amplification and Hybridization of cDNA or cRNA to Illumina Beadarrays

Total RNA from LCM-captured keratinocytes and slices of whole tissue section were extracted using PicoPure RNA isolation kits following the manufacturer’s protocol (Applied Biosystems, #KIT0204). The quality of total RNA was analyzed by Agilent pico chips and RNA with a quality index (RIN) above 5 was used. Total RNA (0.5-1 ng) was then used for cDNA synthesis using the Ovation Pico RNA Amplification System (NuGen, Cat#3302-60). The size distribution of cDNA was analyzed by Agilent Technologies nano chips and the amplified cDNA had a Gaussian distribution with an average size of 200 bp. The cDNA was biotin-labeled per the NuGEN protocol and labeled cDNA (750 ng) was hybridized to Illumina HT-12 beadarrays at the Shared Resource Genome Center at Fred Hutchison Cancer Research Center per the manufacturer’s instructions. The transcriptional data for laser-captured keratinocytes and human genital skin biopsies were deposited in the National Center for Biotechnology Information’s Gene Expression Omnibus (accession No. GSE98540 and GSE172423, respectively).

For cultured keratinocytes, the total RNA extraction, cRNA amplification and hybridization of labeled cRNA to Illumina beadarrays were conducted as previously described (23). Primary human keratinocytes were mock treated or pretreated with IFN-γ at 100 U/ml for 48 hours and such cells were mock infected or infected with HG52 at MOI=1 for 4 hours. Duplicate samples were generated for each condition (untreated + mock infected, IFN-γ treated + mock infected, untreated + HG52 infected, IFN-γ treated + HG52 infected). The array data for keratinocytes mock-treated or IFN-γ-treated, with or without HG52 infection were deposited in the National Center for Biotechnology Information’s Gene Expression Omnibus (accession No. GSE172424).

### Analysis of Beadarray Data

For LCM captured basal keratinocytes from five post-healed skin biopsies and matched arm control skin, raw data were imported to GenomeStudio V2010.3, Illumina (GenomeStudio, RRID: SCR_010973). Control summaries were generated to analyze the quality of hybridization. Data passing this initial quality control step were normalized using Cubic Spline with background subtraction. Normalized data were exported to R and differentially expressed genes were defined as following: three out the five samples have more than three-fold changes over their matched control samples using gene filter package. The differentially expressed genes were analyzed with an unsupervised hierarchical clustering method (Clustering method: UPGMA [weighted average] and similarity measure: euclidean distance) using SpotFire DecisionSite for functional genomics (Spotfire, RRID_SCR_008858). Enriched functional categories and network analyses for differentially expressed genes were performed using Ingenuity Pathway Analysis (IPA 8.8, RRID : SCR_008653). Multiplicity adjustment was performed by controlling false discovery rate. The GoMiner program was used to annotate all the 20,818 genes on Illumina Human HT-12 beadarrays. An annotation database was constructed in Microsoft Access using exported tables from GoMiner.

For primary human keratinocytes, individual samples were normalized by lumi package in R and limma package in R were used to identify differentially expressed genes between untreated + mock infected and IFN-γ treated + mock infected or between untreated + mock infected and untreated + HG52 infected.

### Immunofluorescence Staining

Fresh frozen skin biopsy tissues were cryo-sectioned into 8 µm slices and then fixed and permeabilized in acetone for 20 min at -20°C. After air dry and PBS wash, the slides were incubated with primary antibodies overnight at 4°C and followed by PBS wash, 1-hour incubation with fluorescence-labeled secondary antibodies at room temperature, PBS wash, counterstained with DAPI (Thermo Fisher Scientific Cat# D3571, RRID: AB_2307445) and mounted in Prolong Gold Antifade Mountant (Thermo Fisher Scientific, Cat# P36930). The primary antibodies used in this study were specific for human NCAM (BioLegend Cat# 304602, RRID: AB_314444), CD8 (BD Biosciences Cat# 557708, RRID: AB_314070), CD4 (BioLegend Cat# 300502, RRID: AB_314070), IFI16 (Abcam Cat# ab55328, RRID: AB_2121692), HSV2 (Agilent Cat# B0116, RRID: AB_2335703).

### Fluorescence *In Situ* Hybridization

For human skin tissues, fresh frozen skin biopsies were cryosectioned into 10 µm sections, fixed with chilled 10% buffered formalin and dehydrated in ethanol series. For cultured keratinocytes, cells were fixed with 10% buffered formalin at room temperature, washed with PBS and dehydrated in ethanol series. Dehydrated slides were pretreated with protease K and hybridized using RNAscope multiplex fluorescent assay (Advanced Cell Diagnostics, Cat# 320850), according to the manufacturer’s instruction. The probes used were human CD8A (Advanced Cell Diagnostics, Cat# 310501-C2), IFNG (Advanced Cell Diagnostics, Cat# 560391-C3), IFI16 (Advanced Cell Diagnostics, Cat# 440101), pooled intermediate-early (IE) genes, UL54 (Advanced Cell Diagnostics, Cat# 440131), US1(Advanced Cell Diagnostics, Cat# 440141), US12(Advanced Cell Diagnostics, Cat# 440191), RL2(Advanced Cell Diagnostics, Cat# 440171) and RS1(Advanced Cell Diagnostics, Cat# 440181), positive control PPIB (Advanced Cell Diagnostics, Cat# 313901), and negative control DapB (Advanced Cell Diagnostics, Cat# 310043).

### TCR Reconstruction and Functional Analysis

The T cell receptor (TCR) is encoded by hypervariable *TRA* and *TRB* genes. In order to determine if TCRs that were present in the DEJ CD8TRM were HSV-specific, we first sequenced these genes, then expressed them in reporter cells, and then functionally evaluated these candidate TCRs for recognition of HSV-2, including determination of fine specificity. Genomic DNA from DEJ CD8TRM LCM specimens were sequenced at *TRB* and *TRA* loci by Adaptive Biotechnology (Seattle, WA). The sequencing focused on the V gene segments and the hypervariable CDR3 domains. Sequencing techniques and results have been published (11, 27). For TCR expression in reporter CD8 T cells, full-length, codon-optimized, *TRA* and *TRB* genes were synthesized (Genscript, Piscataway, NJ) with modifications as described (28). Briefly, the gene for *TRB* preceded a P2A sequence for proteolytic separation of the encoded polypeptides, followed by in-frame *TRA*. This was inserted into the lentiviral vector pRRLSIN.cPPT.MSCV/GFP.WPRE (29) with confirmation by sequencing. In this construct, the human *TRA* and *TRB* constant regions were partially replaced with murine homologs with added cysteine residues. This enabled detection and monitoring of reporter cell transduction efficiency with an antibody specific for the murine constant region. Lentiviral particles were made as described (28). Autologous CD8 T cells isolated from PBMC were prepared by negative selection (Miltenyi, San Diego, CA) and activated with anti-CD3 and anti-CD28 beads (Miltenyi). The expression level of the exogenous TCR being studied was measured with anti-mouse TCRβ-PE (H57-597; eBioscience, San Diego, CA) (28) using flow cytometry. TCR-transduced T cells were expanded using anti-CD3 mAb, feeder cells and cytokines as described (28), cryopreserved in aliquots, and thawed and used immediately for assays.

CD8^+^ T cells transduced with exogenous candidate HSV-2-specific transgenic TCRs (tgTCRs) were assessed for recognition of whole HSV-2. To do this, EBV-LCL autologous to the TCR donor were infected with HSV-2 strain 186 at MOI 10 and plated at 2 X 10^4^ cells/well in 96-well U bottom plates. tgTCR-transduced effector cells were added at defined E:T ratios and reactivity was determined by supernatant IFN-*γ* secretion measured by ELISA (30). Previously defined HSV-2-specific CD8 T cell clone 2.1 (31) served as a positive control. Once reactivity to whole HSV-2 was established, published methods (32) used to determine antigenic HSV-1 open reading frame(s) (ORFs) and peptide epitopes were modified for HSV-2. In brief, each HSV-2 ORF from HSV-2 186 (Genbank JX112656.1) was PCR-amplified and cloned into a Gateway™ donor vector (Invitrogen). The HSV-2 genes were then shuttled using Gateway Clonase™ reactions into pDEST103. The pDEST103 plasmid (31) features a CMV promoter directing expression of the gene of interest as a fusion with eGFP. HSV-2 PCR primers, clone identities, and shuttling strategies are published (33). Subject-specific HLA A and B cDNA were PCR-amplified and cloned into pCDNA3.1 as described (30). Sequencing of HLA cDNA genes confirmed match to IMGT (34). To assay which HSV-2 gene and which HLA allele were recognized by tgTCR-expressing reporter cells, we co-transfected Cos-7 cells with each HLA A or B cDNA, and each HSV-2 gene, in duplicate in 96-well plates as described (31). After two days, 10^5^ tgTCR reporter cells were added and activation was detected by IFN-γ secretion. For reporter cells transfected with tgTCR 5491.3, a PE-labeled tetramer (31) containing HLA B*07:02 and a peptide in the HSV-2 protein encoded by gene *UL49* was tested for binding by flow cytometry.

### Statistical Analysis

Two-tailed, paired Student’s t-tests were used in examination of potential differences in expression levels of interferon and HSV viral genes and cell densities of CD8^+^ T cells in biopsy tissue. *P* and *n* values are indicated in the text and Figure legends. For IMA signature gene expression between control skin relative to post-healed skin at the site of prior HSV recurrence at week 2 and again at week 8 post-healing, we used paired t-test and performed multiplicity adjustment using the method of Li and Ji (35), which is more powerful than Benjamini and Hochberg (36) in that it accounts for the effective number of unique genes by computing their correlation. For experiments in the IFI16 knockout fibroblasts, data analysis performed using GraphPad Prism version 8.0, with yields analyzed by two-way ANOVA and Holm-Sidak’s multiple comparisons test (α = 0.05).

## Results

### Keratinocytes Neighboring CD8TRM Show Augmented Antiviral Transcriptional Profiles

**Figure 1:**
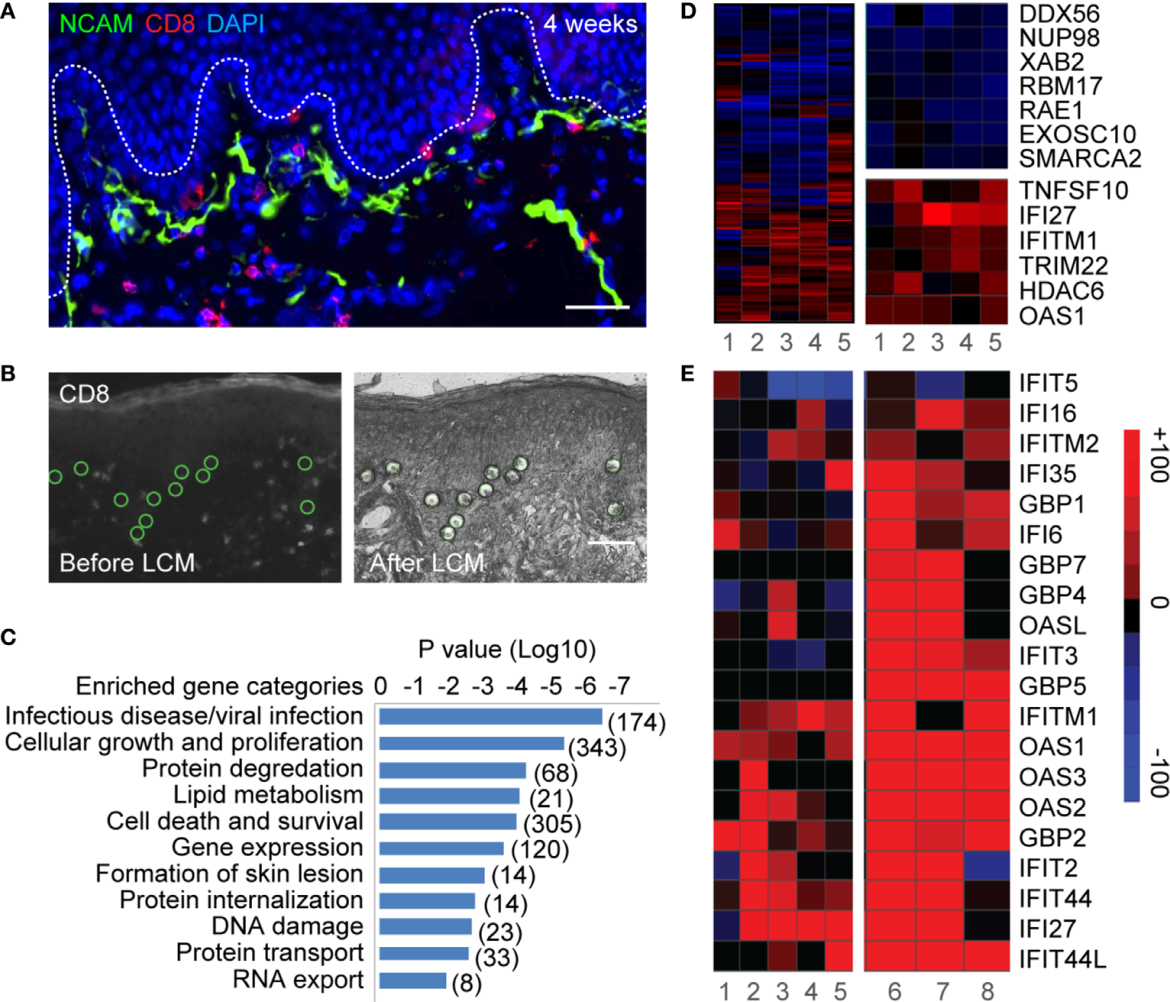
Laser capture microdissection and transcriptional profiling of keratinocytes neighboring CD8TRM in human genital HSV-2 reactivation. **(A)** Innervating nerve endings (green) and CD8^+^ T cells (red) are interconnected with basal keratinocytes at dermal-epidermal junction (DEJ) in a human genital skin biopsy obtained 4 weeks post-healing. Scale bar, 50 µm. **(B)** Micrographs depicting selection (left) and isolation (right) of individual basal keratinocytes in the vicinity of DEJ CD8TRM cells using laser capture microdissection. Scale bar, 50 µm. **(C)** Significantly enriched functional categories of differentially expressed genes in keratinocytes isolated from n = 5 post-healing biopsies compared to matched controls. **(D)** Hierarchical clustering of a set of 174 genes annotated to infectious disease/viral infection (left). Down-regulated genes involved in RNA transcription, processing and transportation (right top) and up-regulated genes annotated to interferon and antiviral responses (right bottom). **(E)** Up-regulation of a subset of ISGs in keratinocytes isolated from post healing skin (n = 5, samples 1-5) and matched HSV-2 lesion (n = 3, samples 6-8).

Keratinocytes and epithelial cells, the major structural components of skin and mucosa, are interconnected with innervating sensory nerve endings, and thus are peripheral targets of both HSV invasion and host defense. DEJ CD8TRM cells interact directly with basal keratinocytes and epithelial cells, to which reactivated HSV-2 viral particles from sensory nerve termini are released (**Figure 1A**). We utilized laser capture microdissection (LCM) and transcriptional profiling to investigate *in vivo* interactions between DEJ CD8TRM and the neighboring keratinocytes. Individual basal keratinocytes were microdissected in the vicinity of DEJ CD8TRM from skin tissue obtained at a post-healing site of HSV-2 recurrence as well as from control arm skin of the same individuals (n *=* 5) (**Figure 1B**, **Supplementary Figure 1** and **Table 1**). We identified 1264 differentially expressed genes, including 569 up-regulated and 695 down-regulated genes, representing 6.1% out of 20,818 genes assessed, in keratinocytes at the sites of prior reactivation compared to those of matched normal controls (**Supplementary Figure 2A**). Functional annotation analysis indicated that genes involved in viral infection in the infectious disease category were the most significantly enriched (P<10^-6^) **(****Figure 1C****)**. Differentially expressed genes in the viral infection category (174 genes, **Figure 1D**) indicated that keratinocytes had down-regulated genes involved in host gene transcription (*DDX56, SMARCA2*) (37, 38) and RNA transportation and processing (*NUP98, RAE1, XAB2, RBM17, EXOSC10*) (39–41). Further network analysis confirmed down-regulation of genes involved in transcriptional machinery (**Supplementary Figure 2B**). Up-regulated genes were related to interferon antiviral activities (*IFI27, IFITM1, TRIM22*, and *OAS1*), caspase activity (*TNFSF10)*, and epigenetic repression (*HDAC6*) **(****Figure 1D****)**. Network analysis identified an enrichment of genes that are shared among type I and type II interferon response pathways (**Supplementary Figure 2C**). The commonly up-regulated genes included the interferon master regulator *IRF3* and other interferon stimulated genes (ISG), such as *IFI27, IFITM1, TNFSF10, IFNAR1, CCL5, HAVCR2* (*TIM3*) and *FGFR4*. Among the annotated ISGs, *IFITM1, IFITM2, IFIT2, IFIT27, IFIT44, IFIT44L, OAS1, OAS2, OAS3, OASL, GBP1, GBP4, IFI6, IFI16*, *IFI27* and *IFI35* were up-regulated in keratinocytes from sites of prior HSV-2 recurrence as well as from active genital herpes lesions (**Figure 1E**), while *GBP5, GBP7* and *IFIT3* were elevated only in keratinocytes from lesion tissues. The ISG expression pattern suggested that multiple antiviral pathways might engage in a cascade fashion in response to the level of infection. Taken together, the LCM-based whole genome transcriptomic analysis argues that keratinocytes neighboring DEJ CD8TRM acquire a robust intracellular antiviral defense mechanism to increase interferon-related responses and decrease host RNA transcription and processing.

### IFI16 Expression Is Widespread and Heightened in Protected Skin Epidermis

**Figure 2:**
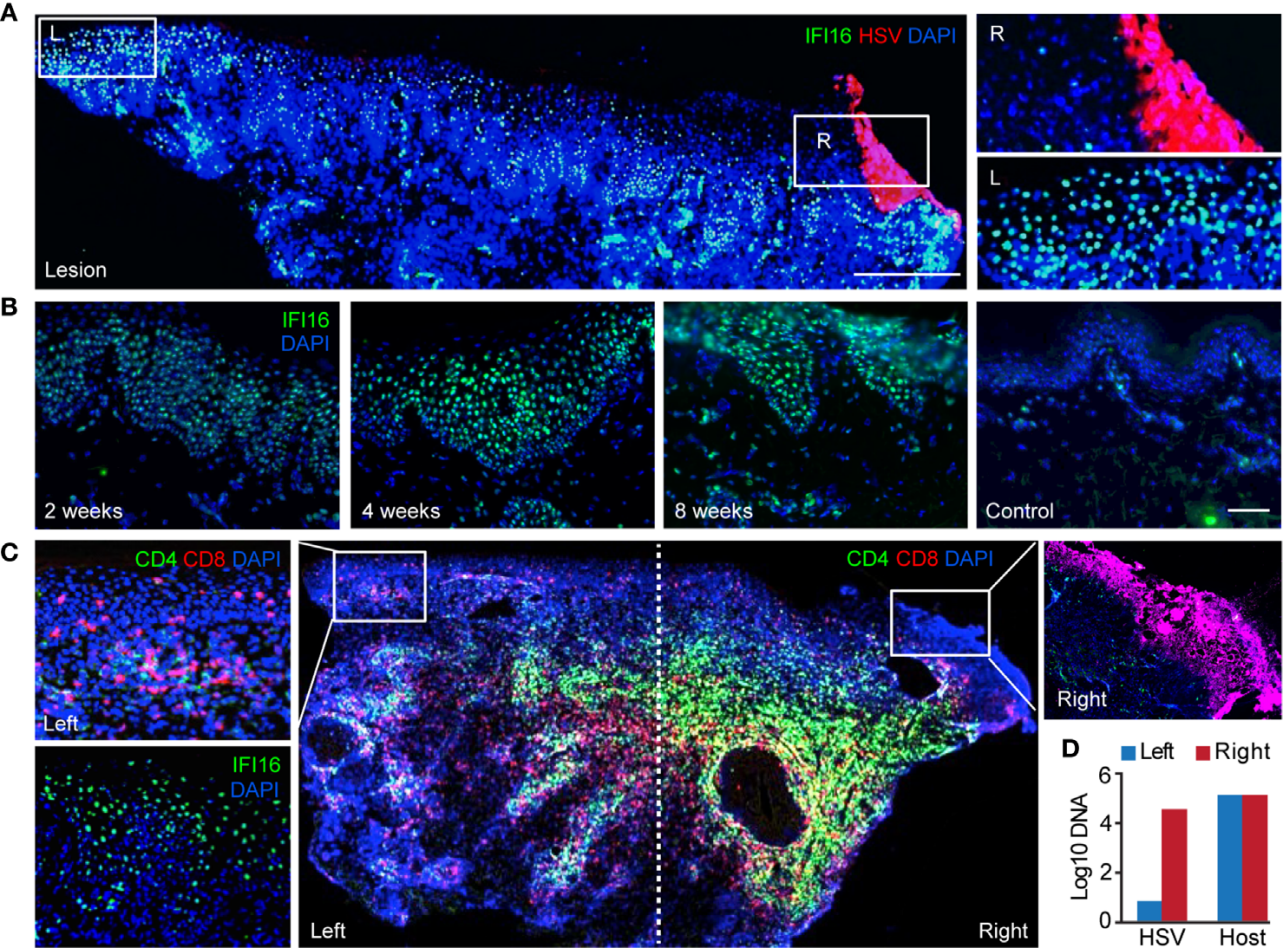
Widespread expression of IFI16 in genital skin during and after HSV-2 recurrence. **(A)** IFI16 expression in a representative HSV-2 ulcer lesion. Right box depicting actively infected epidermis with HSV-2 antigen expression. Left box showing uninfected epidermis distal to HSV lesion. Scale bars, 500 µm. **(B)** IFI16 expression in a representative serial skin biopsies sequentially obtained at 2-, 4-, and 8-week post-healing and in matched arm control skin. Scale bars, 100 µm. **(C)** Distribution of CD4^+^ and CD8^+^ T cells and IFI16 expression in an ulcerative HSV-2 lesion. IFI16 and HSV-2 antigen expression staining were performed on adjacent tissue sections. Inserts are higher magnification of the indicated boxed areas. **(D)** Quantitative PCR measurement of HSV-2 DNA and host genome in the right and left portion of the lesion tissue as designated in **(C)**.

We next evaluated the spatial distribution and temporal expression of antiviral ISGs in relation to CD8TRMs. Interferon gamma-inducible protein 16 (IFI16), known for playing roles in sensing viral infection and suppressing HSV gene expression in cultured cells (42–45) was evaluated as the prototype interferon-related response in genital skin during and after HSV-2 reactivation. IFI16 protein was widely expressed in the epidermis of HSV-2 ulcer-bearing skin (*n* = 6) and localized exclusively in the cell nucleus (**Figure 2A**, inset image L), consistent with its functions in DNA binding and transcriptional regulation (46). Even weeks after lesion resolution, heightened IFI16 expression was evident in large areas of post-healed tissue, but rare in contralateral normal controls (**Figure 2B**). Surprisingly, high-level expression of IFI16 was detected in keratinocytes of histologically normal epidermal tissue, distal to the region of overt viral replication and skin ulcerations (**Figures 2A, C**). High viral genome copies and the detection of viral antigens were accompanied by the absence of detectable IFI16 in HSV-infected cells (**Figures 2C, D**). These *in vivo* observations supported the notion that HSV targets IFI16 for degradation during productive infection (44). Robust IFI16 expression was present in protected epidermal tissue, where rich CD4^+^ and CD8^+^ T-cell infiltration were detected (**Figure 2C**). Thus, tissue-wide innate antiviral gene expression in protected skin epidermis was not related to widespread HSV replication.

### IFN-*γ via* CD8TRM Is the Main Source of Interferon in Skin Epidermis

**Figure 3:**
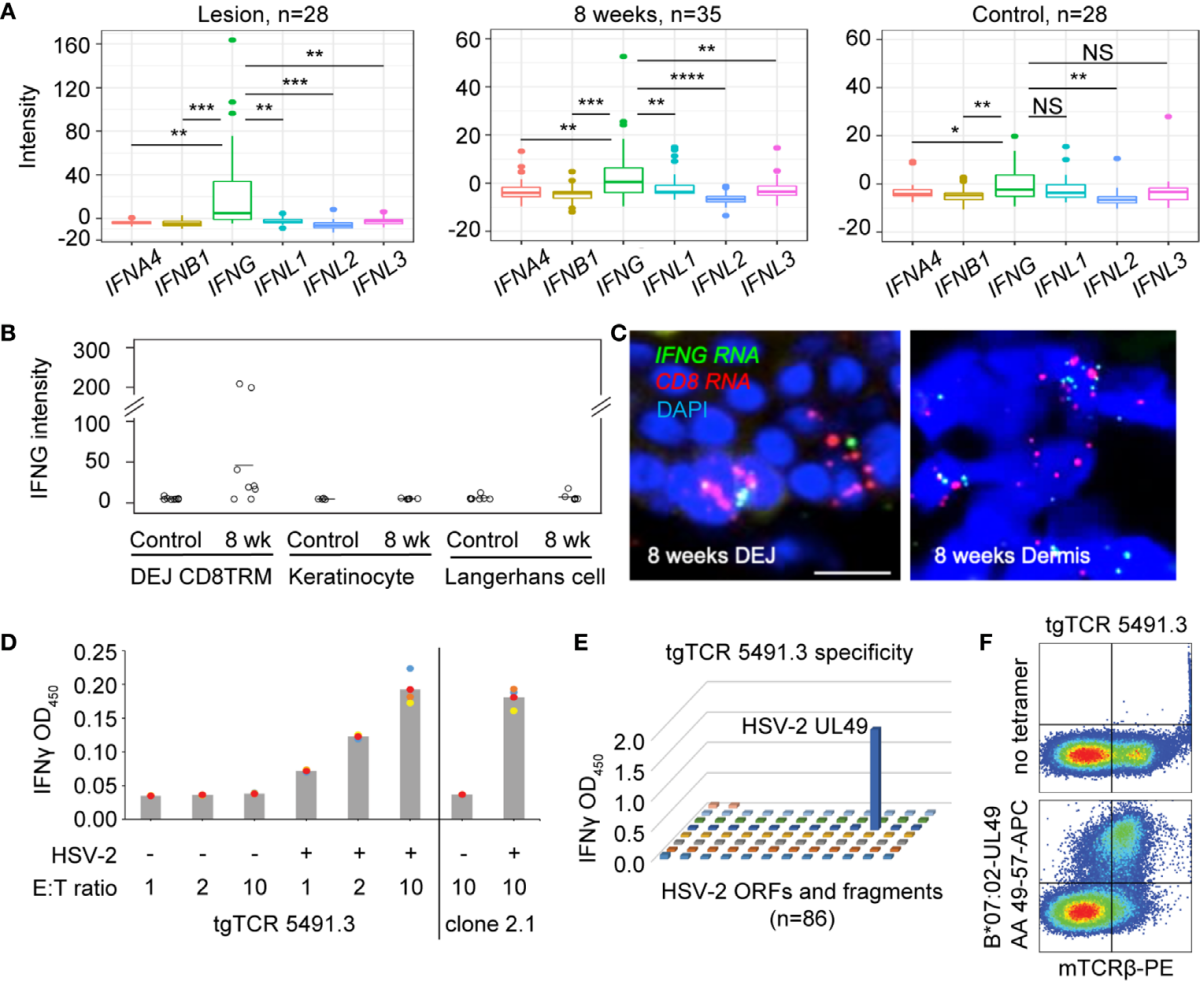
Expression of *IFNG* predominates in HSV-affected skin during and after viral recurrence. **(A)** Transcriptional analysis showing expression levels of *IFN4A, IFNB1* (type I IFN), *IFNG* (type II IFN), *IFNL1, IFNL2* and *IFNL3* (type III IFN) gene in skin biopsies of n = 28 at the time of HSV-2 lesion, n = 35 at the time of 8-week post-healing, and *n* = 28 matched contralateral normal controls. In box plots, the center line is the median, box edges show upper and lower quartiles, whiskers represent the range and dots indicate outliers. ^*^p < 0.05, ^**^p < 0.01, ^***^p < 0.001, ^****^p < 0.0001, NS, not significant; student t test. **(B)** *IFNG* gene expression in DEJ CD8TRM (n = 8), keratinocytes (n = 5) and Langerhans cells (n = 7) laser microdissected from skin biopsies obtained at 8-week post-healing and their matched normal control skin, profiled by whole genome array. **(C)** RNA FISH images depicting co-expression of *IFNG* and *CD8* mRNA transcript in cells (nuclei stained with DAPI) at the DEJ and in the dermis of an 8 weeks post-healing skin biopsy. Scale bars, 20 µm. **(D)** Recognition of autologous HSV-2-infected EBV-LCL by reconstructed transgenic TCR (tgTCR) 5491.3 reporter cells. Effectors and APC were co-incubated at indicated ratios for 24 hours. T cell activation as detected by IFN-γ release. HSV-2-specific CD8 T cell clone 2.1 as positive control. All combinations of effector cells and APC were run in quadruplicate. Each dot an individual value and bars as mean values. **(E)** Representative raw data from HSV-2 proteome-wide screen. Cos-7 cells were co-transfected with HLA B*07:02 cDNA and 86 individual HSV-2 genes or fragments. tgTCR5491.3 reporter cells were added for 24 h and IFN-γ release measured by ELISA. **(F)** tgTCR5491.3 reporter cells stained with anti-mouse TCR beta and tetrameric HLA B*07:02-HSV-2 UL49 AA 49-57 or no tetramer.

To understand the type(s) of interferons that were primarily responsible for increased expression of antiviral genes in HSV-2 affected skin, we compared the levels of various types of interferons expressed in active lesions (n = 28), post-healed tissue at week 8 (n = 35), and contralateral control (n = 28) biopsies (**Supplementary Table 1**). Transcriptional analysis of biopsy tissue indicated that type II interferon, *IFNG*, not type I (*IFNA4* and *IFNB1*) nor type III (*IFNL1, IFNL2, IFNL3*) interferons, was the prevalent interferon gene detected in ulcer lesions and 8 weeks post-healed skin (**Figure 3A**). *IFNG* gene expression was also more dominant than *IFNA4, IFNB1* and *IFNL2* in contralateral normal control skin. This was consistent with previous findings that type I interferon gene transcripts were mostly undetectable in HSV-affected tissues obtained at the time of active ulceration, after healing, and from normal control skin (23).

We delineated the cellular source of *IFNG* in epidermal tissue after lesion healing by performing LCM to obtain individual DEJ CD8TRM, CD1a^+^ Langerhans cells, and basal keratinocytes. We consistently detected *IFNG* transcript expression in CD8TRM located in the DEJ 8 weeks after healing of symptomatic HSV-2 recurrence, but not in uninvolved contralateral control skin obtained on the same day (**Figure 3B**). Fluorescence *in situ* hybridization (FISH) showed co-expression of *IFNG* and *CD8* mRNA transcripts in cells localized at the DEJ and in the deep dermis near blood vessels in skin biopsies obtained at 8-week post-healing (**Figure 3C**), while *PPIB* and *DapB* transcripts showed positive and negative control expression, respectively (**Supplementary Figure 3**). *IFNG* was not detected in the neighboring keratinocytes and Langerhans cells in either the HSV affected or unaffected skin (**Figure 3B**). We investigated CD8TRM cells for HSV-2-reactivity *via* TCR reconstruction and functional assays using tissue from a study subject who has participated in multiple biopsy studies (Material Methods). We observed that DEJ CD8TRM include cells that recognize HSV-2-infected cells and confirmed HSV-2 specificity to a discrete peptide within the protein encoded by HSV-2 UL49 and HLA class I restriction using whole virus proteome screens and tetramer staining (**Figures 3D–F**). Thus, data indicated that DEJ CD8TRM were the major cellular sources of IFN-γ in these tissue samples, that these cells included HSV-2-specific CD8 T cells, and that this cell population was potentially responsible for the widespread and heightened antiviral responses in the surrounding skin epidermis.

### IFN-*γ*-Mediated Intracellular Antiviral Gene Signature in Primary Human Skin Cells

**Figure 4:**
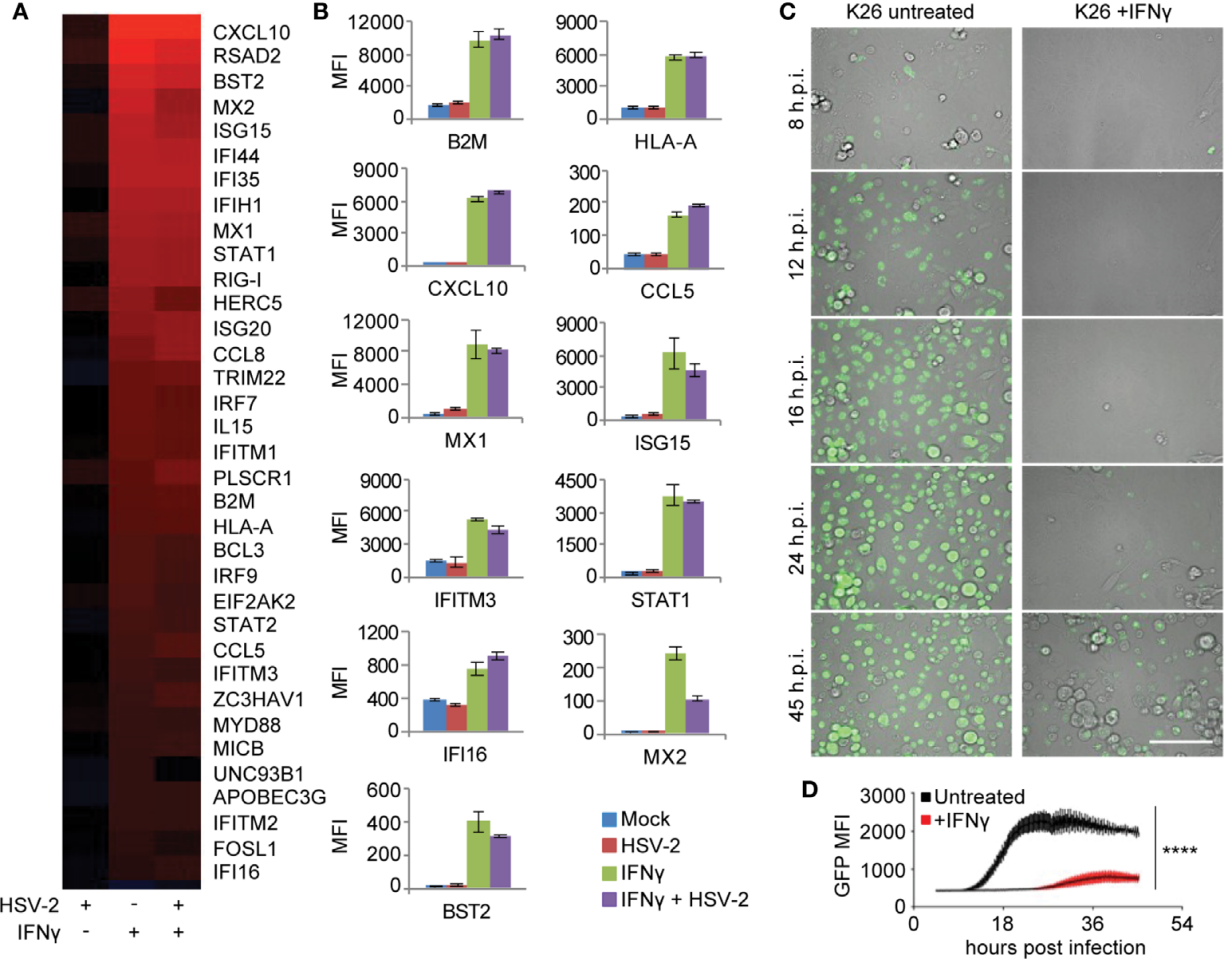
IFN-*γ* mediated antiviral (IMA) gene signature expression in primary human keratinocytes. **(A)** Signature genes differentially expressed by IFN-γ treatment annotated to the GO term “response to virus” in primary cultured human keratinocytes. Column 1: Mock treated, HSV-2 infected, HG52 MOI=1, 4 h.p.i. Column 2: IFN-γ treated, 100 U/ml for 48 hours and then mock infected. Column 3: IFN-γ treated, 100 U/ml for 48 hours and then infected with HG52, MOI=1, 4 h.p.i. Analyses were done by comparing to mock-treated and mock-infected keratinocytes. All genes listed are significantly induced by IFN-γ (FDR = 0.05). False discovery rates were derived from analysis of duplicate samples using Limma package in R. **(B)** Representative host gene expression in the functional category of antigen presentation, chemotaxis, and innate/cell-intrinsic antiviral defense. **(C)** Real-time monitoring of GFP expression in K26-infected primary human keratinocytes (MOI = 1, 45 h.p.i.), mock-treated or treated with IFN-γ 100 U/ml, 48 hours. Scale bars, 100 µm. **(D)** Comparison of GFP signal intensity in K26-infected primary human keratinocytes with or without IFN-*γ* treatment. ^****^p < 0.0001.

To elucidate the role of IFN-γ in CD8TRM mediated immune responses to the tissue microenvironment, we first analyzed IFN-γ mediated effects on primary human keratinocytes, with or without HSV-2 infection. Whole genome transcriptional analyses identified gene categories significantly enriched by IFN-γ pretreatment of keratinocytes (FDR≤0.05), which included antigen processing and presentation, inflammation, innate immune response and antiviral responses (**Supplementary Table 2**). Among the 35 up-regulated genes in the category of ‘response to virus’, genes in antigen presentation (beta-2 microglobulin (*B2M*), *HLA-A* and *MICB)* and chemotaxis (*CXCL10, CCL5* and *CCL8)* were robustly induced by IFN-γ, along with genes implicated in cell-intrinsic antiviral restriction, such as *IFITM1, IFITM2, IFITM3, MX1, MX2, ISG15, HERC5, STAT1, IFI16, BST2* and *RIG-I* (**Figures 4A, B**). HSV-2 infection itself resulted in minimal induction in the antiviral signature gene expression. Instead, HSV-2 infection was shown to have an antagonistic effect on a subset of IFN-γ stimulated genes, such as *MX2, ISG15, HERC5*, and *UNC93B1* (**Figure 4A**), consistent with a large body of literature concerning immune evasion by HSV for type I interferon pathways (47, 48). Thus, this IFN-*γ* mediated antiviral (IMA) gene signature suggests that IFN-*γ* induces a broad array of pathogen restriction factors for innate and cell-intrinsic antiviral defense, in addition to its role in orchestrating and enhancing the adaptive immune responses.

We therefore wanted to assess IFN-γ antiviral activity in primary cultured human keratinocytes and dermal fibroblasts. Live-cell monitoring of keratinocytes infected with K26, a VP26-GFP recombinant HSV-1 strain (49), showed a >40-fold reduction and >16 hours delay in peak GFP expression in IFN-γ pretreated keratinocytes compared to untreated cells (*P <*0.0001) (**Figures 4C, D**). The inhibition of VP26-GFP expression by IFN-γ was also observed in primary human dermal fibroblasts from healthy donors (**Supplementary Figure 4**). The significant delay and reduction of viral gene expression in IFN-γ treated cells corroborated the intracellular restriction predicted by the IMA gene signature, which involves both innate and cell-intrinsic antiviral responses.

### Augmented IMA Gene Signature Expression in Tissue Is Linked to IFN-*γ* Expression by DEJ CD8TRM

**Figure 5:**
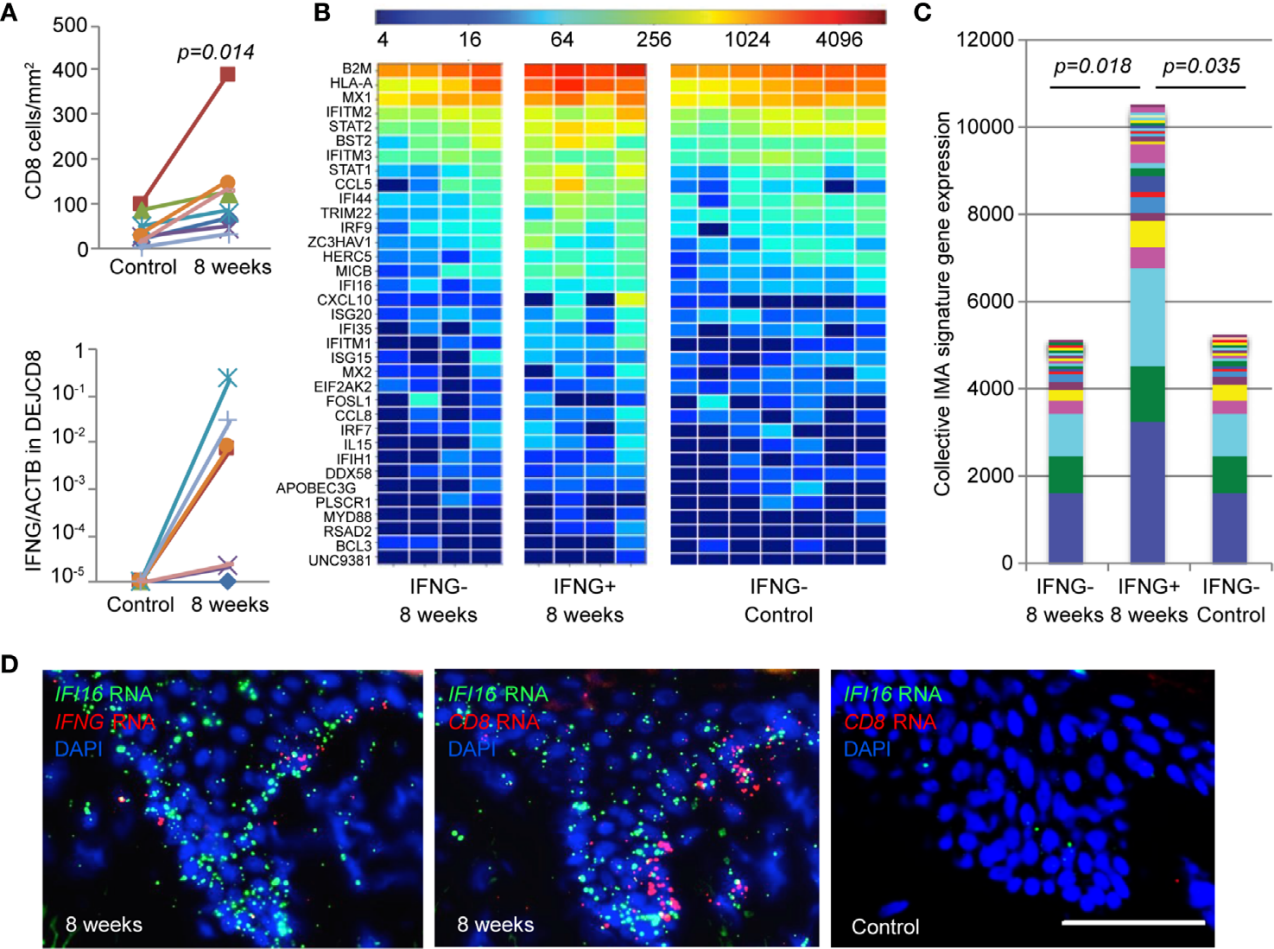
Elevated level of IMA gene signature expression correlates with *IFNG* expression by DEJ CD8TRM cells in HSV-2 post-healing human skin. **(A)** Cell density (upper) and *IFNG* gene expression (lower) of DEJ CD8TRM in skin biopsies taken at the sites of 8-week post-healing and their matched contralateral controls (n = 8). DEJ CD8TRM cells were laser microdissected and coupled with quantitative RT-PCR. *IFNG* gene expression relative to ACTB. Individual **(B)** and collective levels **(C)** of IMA gene signature expression in the 8-week and control set of skin biopsies with or without *IFNG* detection. **(D)** Micrographic images depicting spatial expression of *IFI16*, *CD8* and *IFNG* mRNA transcripts in skin epidermis using RNA FISH dual probe sets of *IFI16* + *IFNG* or *IFI16* + *CD8* in adjacent tissue sections of an 8-week skin biopsy, and dual probe set for *IFI16* + *CD8* on matched normal control skin. Scale bar, 100 µm.

To evaluate the *in vivo* relevance of the IMA gene signature, we first examined the level of *IFNG* expression by DEJ CD8TRM compared with matched tissue-level IMA gene signature expression in skin biopsy tissue (*n* = 8) (**Figure 5**). A significantly higher density of CD8TRM were detected in skin biopsies taken at 8 weeks post-healing of prior HSV-2 recurrences than in uninvolved contralateral control skin collected at the same time (median 106 cells/mm^2^ *versus* 30 cells/mm^2^, respectively) (**Figure 5A**, upper panel). DEJ CD8TRM were individually obtained *via* LCM and assayed for *IFNG* mRNA expression. DEJ CD8TRM from four of the eight biopsies taken at 8 weeks had detectable *IFNG* expression, but not CD8 cells from the contralateral control side (**Figure 5A** lower panel). Skin tissue containing DEJ CD8TRM positive for *IFNG* expression demonstrated both elevated levels and an increased breadth of the IMA signature expression. Collectively, a significant 2-fold increase in IMA gene signature expression was seen in skin tissues containing *IFNG*-expressing CD8TRM compared to normal control skin or post-healed tissue with undetectable *IFNG* (**Figures 5B, C**). These results support the notion that *IFNG* expression by DEJ CD8TRM is associated with enhanced innate and intrinsic antiviral responses at the tissue-level in humans.

To visualize the DEJ CD8TRM, IFN-γ and IMA expression *in situ*, we analyzed *CD8*, *IFNG* and *IFI16* mRNA transcripts in representative 8 weeks post-healing skin biopsies and contralateral control tissue. RNA FISH analysis exemplified the abundant expression of *IFI16* mRNA transcripts in skin epithelium, where expression of *CD8* and *IFNG* mRNA were detected in an 8-week post-healing tissue (**Figure 5D**). In contrast, *IFI16* mRNA appeared scarce in the epidermis of contralateral control skin, where *CD8* mRNA expression was absent. These images depict the spatial relationships between DEJ CD8TRM cells, *IFNG* expression and elevated *IFI16* expression in the epidermis, and support the hypothesis that DEJ CD8TRM, through IFN-*γ* production, instruct the neighboring epithelial cells to induce innate antiviral responses in the tissue microenvironment.

### Patterns of IMA Expression in Normal and HSV-Affected Human Skin

**Figure 6:**
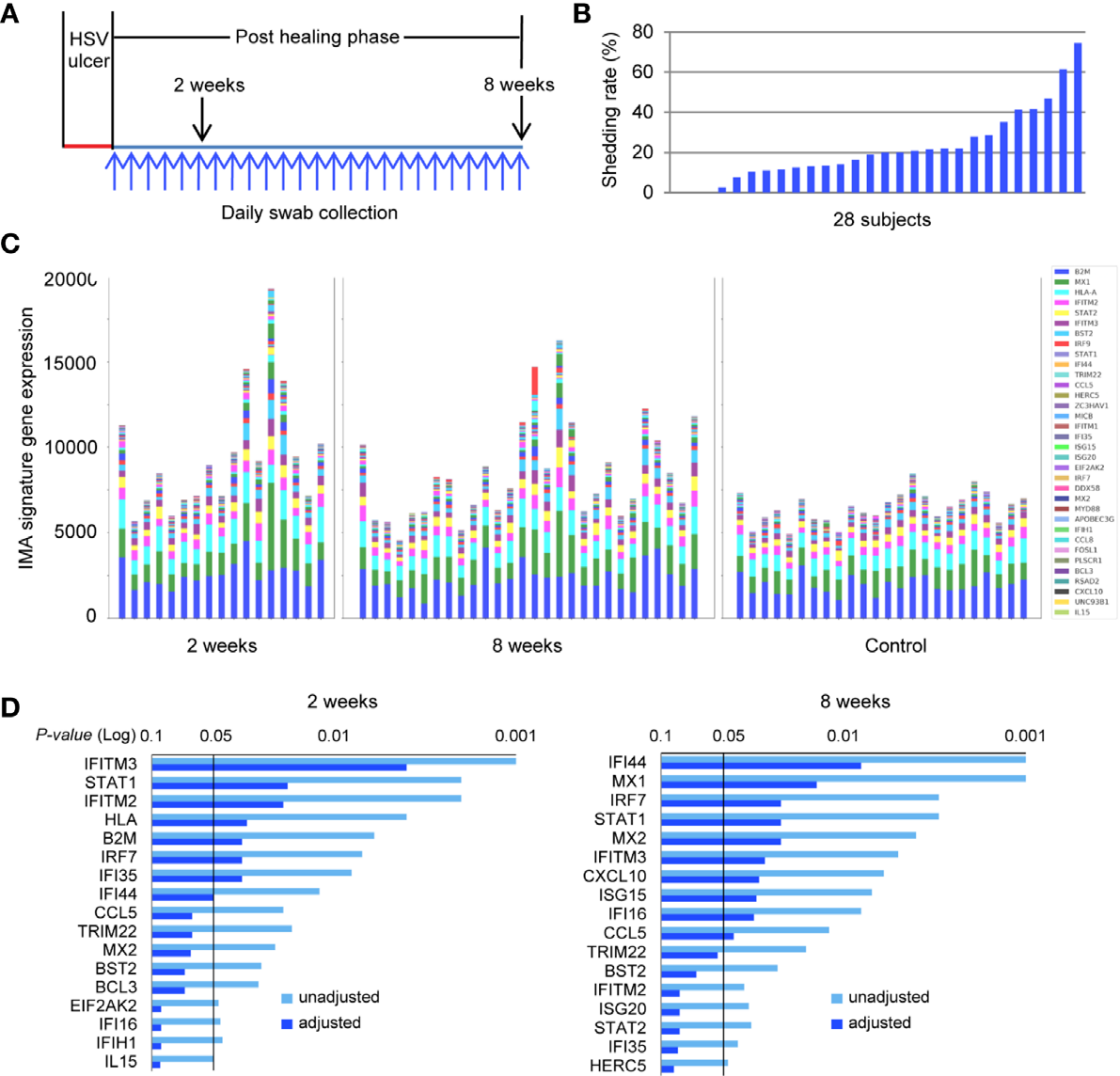
IMA signature expression pattern is distinct between HSV-2-affected and normal skin in a patient cohort across a wide spectrum of genital herpes outcomes. **(A)** Study design. Genitalia HSV-2 reactivation was monitored by daily swab collection and PCR detection for an 8-week study period after a clinical symptomatic recurrence. Skin biopsies were obtained sequentially at the original lesion site 2- and 8-week post-healing. A control biopsy was taken at the contralateral normal site at the 8-week timepoint. Daily swabs were collected starting at day 7 after the onset of a lesion-forming HSV-2 reactivation. **(B)** HSV-2 shedding rates were calculated as days of positive HSV DNA detection within the total numbers of days of that swabs were collected during the study period for each participant, n = 28 subjects. **(C)** Skin biopsies were processed for RNA extraction and whole genome transcriptional profiling. The collective levels of IMA gene signature expression in post healing skin taken at week 2 (n = 17) and week 8 (n = 28) of prior HSV-2 recurrence and in normal contralateral control skin (n = 24). **(D)** IMA signature genes significantly associated with sites of prior HSV infection at 2- or 8-week post healing time. Multiplicity adjusted and unadjusted paired t-test.

We investigated further the prevalence of the IMA gene signature in a larger study cohort of 28 subjects who had clinically proven recurrent HSV-2 disease. HSV-2 reactivation was monitored by collecting daily swabs in the genitalia for an 8-week study period, and biopsy tissue was obtained both at the site of the HSV lesion and an uninvolved contralateral control site (**Figure 6A**). HSV DNA detection from the overall set of 1,568 daily swabs ranged, among subjects, from 0 to 74.6% of days positive for HSV during the 8-week study time (**Figure 6B**). Thus, our study cohort has a wide spectrum of viral activity and subclinical expression of HSV-2 reactivation, similar to the spectrum of immune competent subjects that we have studied in the last 20 years (17, 18). Transcriptional profiling of biopsies obtained from uninvolved normal controls (n = 24) and HSV-2 affected sites, at 2- and 8-week post healing (n = 17, n = 28, respectively), exhibited striking differences in IMA gene signature expression (**Figure 6C**). Expressions of genes encoding *B2M, HLA-A*, *MX1, IFITM2, STAT2, IFITM3*, and *BST2*, which are implicated in antigen presentation and cell-intrinsic antiviral immunity, were well-conserved in hierarchy and abundance in all the normal uninvolved skin epithelia, irrespective of their anatomical locations, obtained from labia majora, mons pubis, perianal, perineum, buttock, gluteal cleft and thigh (**Figure 6C** and **Table 1**). Notably, the elevated expression and broadened spectrum of IMA gene signature seen in several post healing tissues resembled closely the IMA expression pattern seen in skin tissue positive for *IFNG* expression by DEJ CD8TRM cells (**Figure 5C**). A subset of IMA signature genes, including those known to participate in innate and intrinsic restriction of viral infection *(IFI16, TRIM22, IFITM2, IFITM3, ISG15, IRF7, STAT1, MX1* and *MX2*), chemokines (*CCL5* and *CXCL10)* and other ISGs (*IFI35, IFI44*), were significantly increased in post-healed skin biopsies at 2- or 8-week post healing (**Figure 6D** and **Supplementary Tables 2, 3**). Thus, the expression pattern of these IMA signature might serve as a surrogate biomarker for enhanced innate immune protection in the barrier tissue.

### IFN-*γ* Blocks HSV Gene Transcription at Immediate-Early Stage and IFI16 Restricts Wild Type HSV-2 Replication

**Figure 7:**
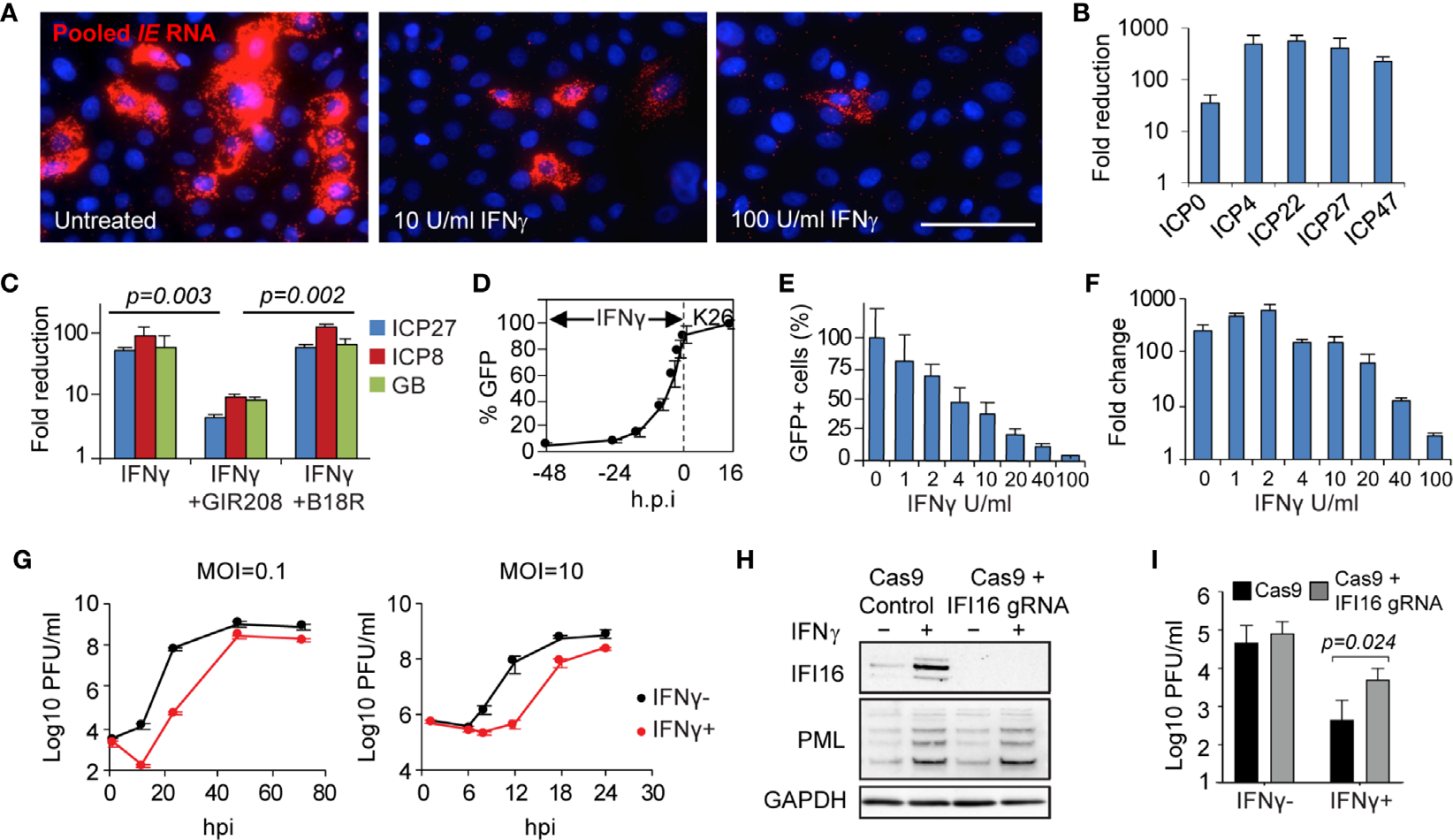
Mechanism, specificity and effectiveness of IFN-*γ*-mediated antiviral action in primary human skin cells. **(A)** Evaluation of IFN-*γ* effect on HSV immediate early (IE) gene transcription *via* RNA FISH. *In situ* detection and visualization of IE gene expression using pooled probes specific to each of the five IE genes. Primary human keratinocytes 6 h.p.i. with K26 (MOI=1) either mock-treated or pretreated with IFN-γ (10 U/ml or 100 U/ml). Scale bar, 50 µm. **(B)** Quantitative assessment of IFN-γ-mediated reduction of IE gene expression *via* qRT-PCR. Primary human fibroblasts infected with wildtype strain KOS, MOI=1, 4 h.p.i. Mock treated compared to IFN-*γ* treated, 100 U/ml. **(C)** IFN-γ effect on HSV gene transcription (ICP27, ICP8, and glycoprotein B) in the presence or absence of interferon blockers, GIR208 and B18R. Primary human keratinocytes infected with KOS, MOI=1, 4 h.p.i. **(D)** Time-dependent inhibition by IFN-*γ*. Percentage of GFP-expressing cells in K26-infected primary human keratinocytes 16 h.p.i. (MOI=1) with IFN-*γ* treatment initiated at 2, 4, 8, 16, 24, or 48 hours prior to infection. **(E, F)** Dose-dependent inhibition by IFN-*γ*. GFP expression **(E)** and viral DNA replication **(F)** under increasing dosage of IFN-γ in K26-infected keratinocytes. MOI=1, 16 h.p.i. HSV genome DNA were quantified by qPCR and compared between 2 and 16 h.p.i. **(G)** Growth kinetics of strain KOS under low and high MOI in primary human fibroblasts with (100 U/ml) or without IFN-γ. **(H)** Responsiveness to IFN-γ in IFI16 knockout and Cas9 control human fibroblasts. Immunoblot of whole-cell lysate from IFI16 knockout and Cas9 control cells mock-treated or pretreated with IFN-γ (500U/mL) for 20 hours. One of three biological replicates is shown. **(I)** Viral yield 12 h.p.i. following wildtype HSV-2 strain 186 infection (MOI=0.1) of IFI16 knockout and Cas9 control fibroblasts mock-treated or pretreated with IFN-γ (500U/mL) for 20 hours. Two-way ANOVA with Holm-Sidak’s multiple comparisons test. Values are mean ± S.D. (*error bars*), from three independent experiments.

The *in vivo* evidence of host transcriptional down regulation shown in keratinocytes (**Figure 1**) led us to examine if IFN-*γ* had an effect on the initial stage of the HSV lifecycle. In IFN-*γ* pretreated primary human keratinocytes and fibroblast cells, viral transcription of all five immediate-early (IE) genes, *ICP0, ICP4, ICP27, ICP22, ICP47*, was substantially reduced as assayed by either RNA FISH detection or real-time RT-PCR quantification (from 30 to >100 fold) (**Figures 7A, B**). IFN-*γ* suppression of all classes of viral gene transcription, as represented by *ICP27, ICP8* and *gB*, required IFN-*γ* receptor signaling (**Figure 7C**). Addition of a neutralizing antibody specific to IFN-*γ* receptor α chain (GIR-208), abolished IFN-*γ* inhibition of HSV gene transcription (*P* = 0.003). Conversely, the type I interferon blocker, B18R, had no effect on IFN-*γ* induced inhibition (*P* = 0.274 for IFN-*γ*, *P* = 0.002 for GIR-208). Further testing on viral gene expression indicated that IFN-*γ* inhibition was effective under a wide range of pretreatment time and dosage, even under short duration (such as 4 hours) or low concentration (such as 4 U/ml) (**Figures 7D, E** and **Supplementary Figure 5**). Quantitative assessment of viral genome copies indicated that IFN-*γ* treatment hindered viral DNA replication, most obviously at a dose of or higher than 20 U/ml/100,000 cells, with 2.5, 13.7 and 75.4-fold reduction at 20, 40 and 100 U/ml of IFN-*γ*, respectively (**Figure 7F**). Thus, IFN-*γ*-mediated inhibition on HSV gene transcription and viral DNA replication takes effect in a time- and dose-dependent manner.

We also investigated the impact of IFN-*γ* on the viral growth kinetics during both low and high multiplicity of infection (MOI) (**Figure 7G**). Multi-step growth curves at low multiplicity of infection (MOI=0.1 pfu/cell) showed >1000-fold reduction of virus titer at 24 h.p.i. in IFN-*γ* pretreated compared to untreated primary fibroblasts. IFN-*γ* delayed virus replication by about 24 hours with regards to reaching titers similar to untreated cells. In single-step growth curves, at high multiplicity of infection (MOI=10 pfu/cell), a prolonged delay from 6 to 12 h.p.i. was noted. The extra lag time in growth kinetics further confirmed that IFN-*γ* treatment blocks the initiation of the virus lifecycle. The additional 6-8 hours delay in viral replication could provide a critical margin needed for the host to orchestrate immune defenses to eliminate the virus before the clinical manifestations developed.

To evaluate if IFI16 would enhance IFN-*γ* ****associated antiviral effects, we compared viral yields with and without IFN-*γ* pretreatment in IFI16 knockout fibroblasts. Immunoblot analysis confirmed the loss of IFI16 expression in the knockout cells, with IFI16 induced by IFN-*γ* only in the Cas9-expressing control cells (**Figure 7H**). Equivalent induction of promyelocytic leukemia protein (PML) expression in the two cell lines indicated that the IFI16 knockout and control fibroblasts were both responsive to IFN-*γ* treatment (**Figure 7H**). Loss of IFI16 in the knockout cells increased the yields of wildtype HSV-2 186 strain by only 1.5-fold relative to control cells in the absence of IFN-*γ* treatment (**Figure 7I**). However, wildtype virus replication was increased by 9-fold in the IFI16 knockout cells relative to control cells following IFN-*γ* treatment (*P*=0.0236), indicating the increased role of IFI16 in suppression of viral replication when induced by IFN-*γ*. Therefore, intracellular antiviral factors such as IFI16 contribute to the restriction of wildtype virus replication and are likely more effective following IFN-*γ* induction.

## Discussion

The mechanisms by which tissue-resident memory cells rapidly initiate an immune response that controls microbial infection within an entire tissue have not been defined in humans. Based on detailed *in situ* dissection, our studies reveal novel immunological crosstalk between CD8TRM and the neighboring tissue microenvironment in human skin and genital mucosa, one in which the adaptive immune response initiates innate/intrinsic antiviral effects in the surrounding epithelial cells. We had previously shown that CD8TRM included cells that bound HSV-2 peptide/HLA multimers *in situ* (10, 11). We now confirm and extend this finding that a portion of CD8TRM is HSV-2-specific using TCR functional reconstruction, yielding reporter cells that recognize both whole virus and a specific HSV-2 peptide. Here, we show that CD8TRM can bridge the adaptive tissue-resident T cell immune response to innate intracellular restriction to block viral replication, *via* cytokine IFN-*γ*; achieving a widespread and effective antiviral host defense. We identified an IMA gene signature in epithelial cells that is indicative of an augmented cell-intrinsic antiviral state and poised for pathogen resistance and restriction. Enhanced IMA gene signature expression was anatomically associated with DEJ CD8TRM enrichment in the protected skin/epithelium. Distinct spatial correlation between low-level and enhanced IFI16 expression, inside and outside the zone of active viral production, respectively, provides evidence *in vivo* for the role of IFI16 in immune resistance to HSV and confirms an evasion strategy of HSV to disarm host innate antiviral responses (47, 48, 50). We further connect these *in vivo* results with cell culture studies showing that IFI16 can contribute to restriction of replication of wildtype HSV-2. Our results highlight the importance of IMA genes in barrier protection against virus spread and disease manifestation. Differences in expression level and functional regulation of IMA signature genes might contribute to the wide spectrum of clinical outcomes observed in human genital herpes infection.

Innate immune responses have traditionally been thought to activate the adaptive immune responses especially during initial, primary infection (1). Our data indicate that there are additional complexities in host-pathogen interactions in chronic, recurring infections and that adaptive immune cells can instruct innate responses during a recurrent infection. We observed that CD8TRM cells recognize viral antigens and produce IFN-*γ*, which induces the expression of several ISGs in surrounding cells, including HSV restriction factors such as IFI16 and TRIM22. IFI16 at basal levels can restrict replication of HSV-1 mutant ICP0^-^ viruses (43), and when overexpressed, it can restrict wildtype HSV-1 (44). Our results connect the induction of IFI16 expression in uninfected cells surrounding herpetic lesion tissue to the ability of IFN-*γ*-mediated elevated levels of IFI16 to restrict wildtype HSV-2 replication in normal human fibroblasts. Thus, IFI16 serves as an intrinsic resistance factor at basal levels that is induced as an innate response to block wildtype viral replication. Another gene product that we observed as elevated in the surrounding uninfected cells is TRIM22, which we recently showed to be a restriction factor for HSV-1 infection (51). Together, our results demonstrate that adaptive immune cells can instruct the innate immune responses in tissue microenvironments to mount mechanisms that restrict viral replication, thus providing evidence of a new feedback loop connecting adaptive and innate immune mechanisms. The results also indicate the *in vivo* overlapping nature of intrinsic resistance, innate immunity, and adaptive immunity.

Induction of a broad spectrum of restriction factors *via* IFN-*γ* treatment blocked viral immediate-early gene transcription, the earliest event in the HSV life cycle in primary human epithelial cells. Hindering viral gene expression resulted in delayed viral growth, reduced viral burden, and increased lag time for an additional 6-8 hours before production of infectious virus. Our prior studies of HSV dynamics have indicated that a lengthening of 40 mins in the average lifespan of infected cells could result in 100-fold difference in HSV production (52). The additional 6-8 hours delay in viral replication could result in ~1000-fold reduction in virus burden, providing the host extra leeway in immune defense that could change the clinical outcomes from an erosion lesion to subclinical reactivation. Overall, our studies reveal a novel immune crosstalk mechanism underlying successful host containment of viral infection through a cytokine mediated communication from CD8TRM that creates an antiviral “field effect” impacting neighboring epithelial cells and beyond.

We have performed transcriptional profiling of laser captured keratinocytes, CD8TRM cells, and Langerhans cells, and while all three cell types express little type I IFN, CD8TRM cells significantly express IFN-γ. CD4^+^ T cells, dendritic cells and macrophages also reside in dermal area (20, 23). CD4^+^ T cells through cytokine IFN-γ expression can provide long-ranged control of intracellular pathogen (53). IFN-γ plays an important role in controlling HSV reactivation (54–56). Several ISGs, such as ISG15 and OAS1, have been shown to assist containment of HSV infection in the murine model (57, 58). Humans with mutations in STAT1, TLR3 or UNC93B are susceptible to HSV encephalitis (59–61). Since TLR3 and UNC93B are involved in TLR mediated type I interferon production and Stat1 is a critical signaling molecule for both type I and type II interferon, these genetic studies provide *in vivo* evidence supporting the critical roles of IFN-*γ* mediated innate immune defense against HSV infection.

We notice that IMA expression in normal genital skin have very similar levels and hierarchical patterns across the patients we have studied. This phenomenon is intriguing and consistent with the literature which suggests that stem cells express a subset of ISGs as their cell intrinsic defense to resist viral infection, and many of these ISGs decrease when they differentiate and become susceptible to viral infection (62). It is tempting to speculate that epidermal keratinocytes in human genital skin constitutively express a subset of ISGs to resist low levels of HSV-2 reactivation and elevate other specific ISGs in response to IFN-γ by TRM to inhibit higher levels of HSV-reactivation. These detailed mechanisms on how a subset of ISGs respond to IFN-γ and control asymptomatic HSV-2 reactivation is of great interest for the future studies. Ultimately, we desire to elucidate the optimal immune responses that lead to successful control in asymptomatic HSV-2 reactivation and design a vaccine strategy to achieve this type of local protection.

Although we studied these immune mechanisms in the context of human recurrent genital herpes infection, the signature genes induced by IFN-*γ* produced by the CD8TRM could also impact other viral infections. Among the ISGs that we observed to be induced, IFITM proteins have been shown to mediate resistance to influenza virus A, West Nile virus, and dengue virus (63) and filoviruses (64). TRIM22 has been observed to restrict herpesviruses (51), hepatitis virus B (65), encephalomyocarditis virus (66), HIV (67, 68) and influenza (69). Finally, IFITM2 can restrict SARS coronavirus 2 infection (70). Thus, tissue-resident memory cells, upon encountering viral antigens in barrier tissue, could produce IFN-*γ* that activates the expression of an array of interferon-stimulated genes in the surrounding cells and tissue that further resist and restrict pathogen spread. Therefore, this mechanism for bridging adaptive immunity to innate immunity could be an important immune process for controlling viral infection in immunized individuals or re-infected individuals and controlling various viral diseases ranging from herpes to influenza, AIDS and even COVID19.

## Data Availability Statement

The datasets presented in this study can be found in online repositories. The names of the repository/repositories and accession number(s) can be found below: https://www.ncbi.nlm.nih.gov/, GSE98540, GSE172423 and GSE172424.

## Ethics Statement

The study protocol was approved by the University of Washington Institutional Review Board. The patients/participants provided their written informed consent to participate in this study.

## Author Contributions

JZ formulated and designed the study, analyzed and interpreted the data, and led the writing of the manuscript. TP designed the study, performed computation analysis and data mining. KP executed laser microdissection and transcriptional array experiments. CS and MM conducted CRISPR knockout cell experiments and viral infection. SS performed RNA FISH experiments and live-cell imaging. YH, EL, LJ, and AK performed *in vitro* tissue culture experiments and biopsy tissue staining. KD performed lenti-vector TCR reconstruction. LCJ and KL contributed to HSV-2 library construction, proteome screening and tetramer staining. DKo provided clinical isolates of primary fibroblasts. AM performed statistical analysis. AL contributed expression data visualization. M-LH and KJ provided HSV DNA detection and quantification. CJ and AW directed human biopsy studies and supervised the clinic. JZ, LC, DKo, and DKn coordinated the study. All authors contributed to the article and approved the submitted version.

## Funding

This work was supported by grants from the National Institutes of Health (AI111780, AI143773, TR003208 to JZ, AI063106, 75N93019C00063 to DKo, AI106934 to Dkn, AI042528, AI134878 to LC, AI030731 to AW) and NIH predoctoral fellowships F31 AI129207 to MM and AI145062 to CS.

## Conflict of Interest

The authors declare that the research was conducted in the absence of any commercial or financial relationships that could be construed as a potential conflict of interest.

The reviewer has declared a shared affiliation with some of the authors CS, MM, DK to the handling Editor at the time of review.

## Publisher’s Note

All claims expressed in this article are solely those of the authors and do not necessarily represent those of their affiliated organizations, or those of the publisher, the editors and the reviewers. Any product that may be evaluated in this article, or claim that may be made by its manufacturer, is not guaranteed or endorsed by the publisher.
