# Supplementary material for: Tissue-Resident-Memory CD8+ T Cells Bridge Innate Immune Responses in Neighboring Epithelial Cells to Control Human Genital Herpes
Source: Front Immunol. 2021 Sep 6;12:735643. doi: 10.3389/fimmu.2021.735643 (PMC8450389; doi:10.3389/fimmu.2021.735643)
Supplement: Supplementary file 1 [file DataSheet_1.pdf]

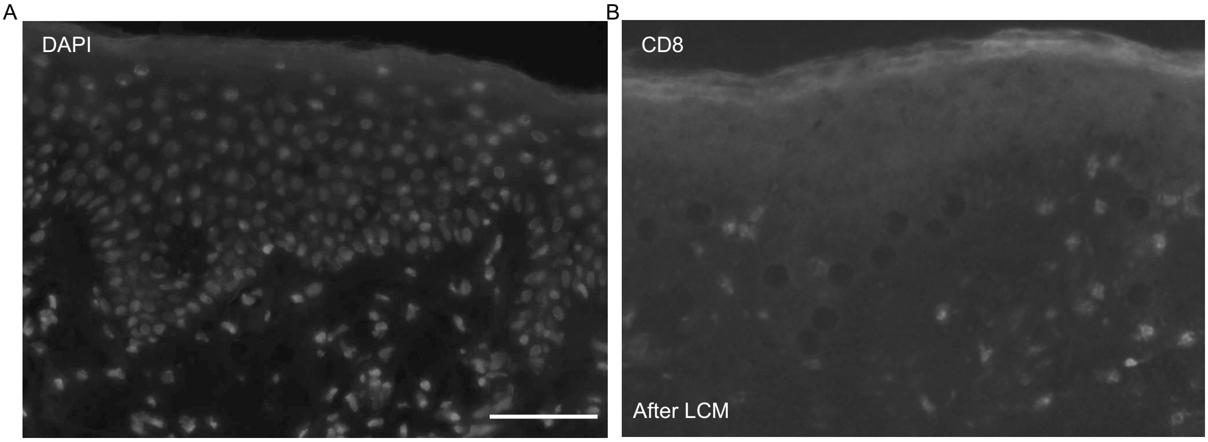

Supplementary Figure 1. **Isolation of basal keratinocytes in skin biopsies at the single cell level by laser capture microdissection.**
(**A**) DAPI nucleus staining depicting the structure of epidermal and dermal layers. Scale bar, 50 µm. (**B**) Captured keratinocytes are free from nearby $CD8^+$ T cells.

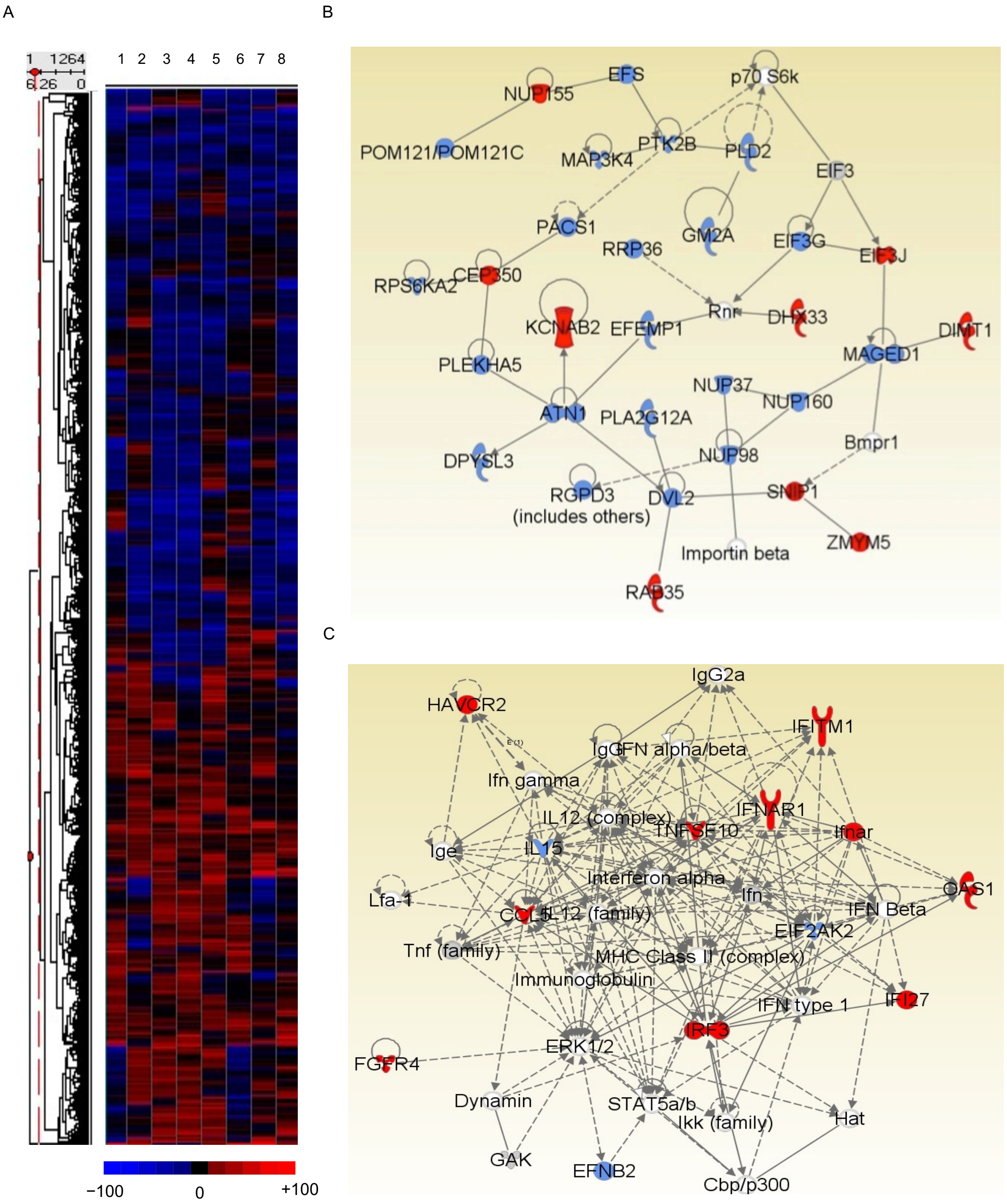
A
B
C
1 1264
6.26 0
1 2 3 4 5 6 7 8
−100
0
+100
NUP155
EFS
p70 S6k
POM121/POM121C
MAP3K4
PTK2B
PLD2
EIF3
PACS1
RRP36
GM2A
EIF3G
EIF3J
RPS6KA2
CEP350
KCNAB2
EFEMP1
Rnr
DHX33
DIMT1
PLEKHA5
MAGED1
NUP37
NUP160
ATN1
PLA2G12A
DPYSL3
NUP98
Bmpr1
RGPD3
(includes others)
DVL2
SNIP1
Importin beta
ZMYM5
RAB35
IgG2a
HAVCR2
IFITM1
IgG
IFN alpha/beta
Ifn gamma
IL12 (complex)
IFNAR1
TNFSF10
Ifnar
Ige
IL15
Interferon alpha
Lfa-1
Ifn
OAS1
CCL5
IL12 (family)
IFN Beta
EIF2AK2
Tnf (family)
MHC Class II (complex)
Immunoglobulin
IFN type 1
IFI27
IRF3
ERK1/2
FGFR4
Dynamin
STAT5a/b
Ikk (family)
Hat
GAK
EFNB2
Cbp/p300

Supplementary Figure 2. **Keratinocytes surrounding the DEJ CD8 T cells down-regulate genes in host transcription, but up-regulate expression of interferon stimulated genes.** (**A**) Hierarchical clustering of differentially expressed genes (1264 genes) through whole genome transcriptional profiling of individually laser microdissected keratinocytes neighboring DEJ CD8^{+} T cells from n = 5 post-healing skin biopsies (1, 2, 3, 4, 5) and from n = 3 lesion biopsies (6, 7, 8) in comparison to their matched normal arm controls. On average, about 100 keratinocytes were captured from each biopsy. (**B**) Pathway analysis showing a group of down regulation of genes involved in host transcriptional machinery. (**C**) Pathway analysis identifying up-regulation of genes shared by type I and type II interferon signaling pathways. The key to different symbol shapes in B&C can be found in https://qiagen.secure.force.com/KnowledgeBase/articles/Basic_Technical_Q_A/Legend

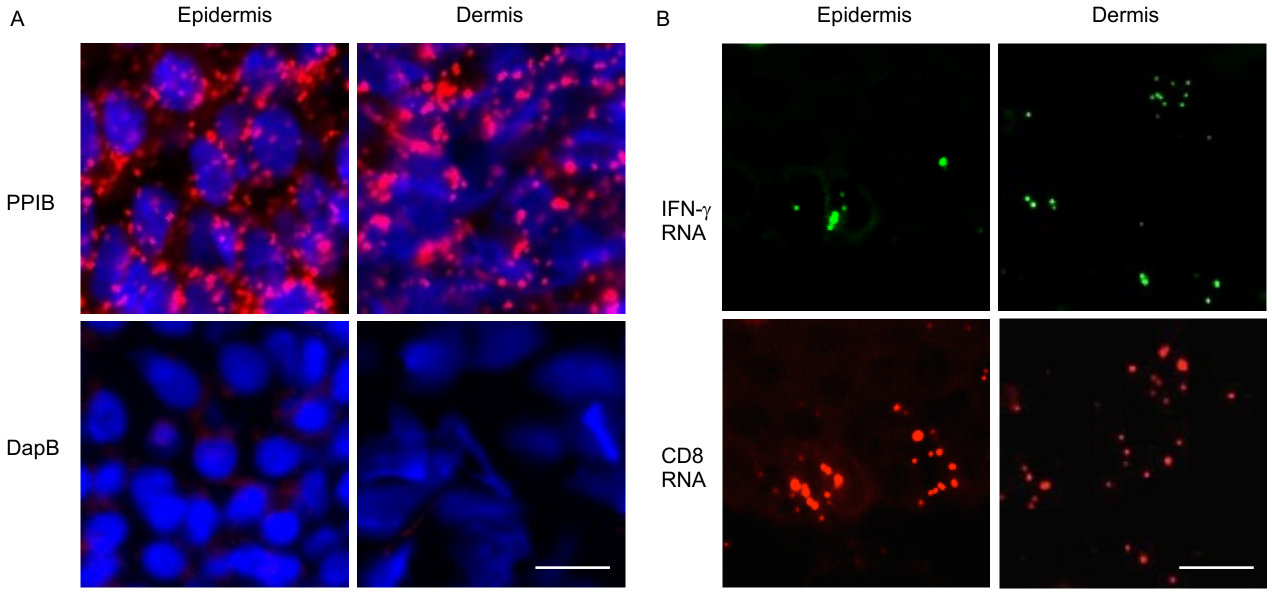

Supplementary Figure 3. **Fluorescence in situ hybridization (FISH) to detect mRNA transcripts in human skin**.
(**A**) Representative micrographs depicting positive (PPIB) and negative (DapB) probe signals in the epidermal and dermal layer of an 8-week post-healing skin biopsy. Scale bars, 20 µm. (**B**) Separation of individual signals of *IFNG* and *CD8* mRNA transcript expression in the dual FISH staining shown in Fig. 3C.

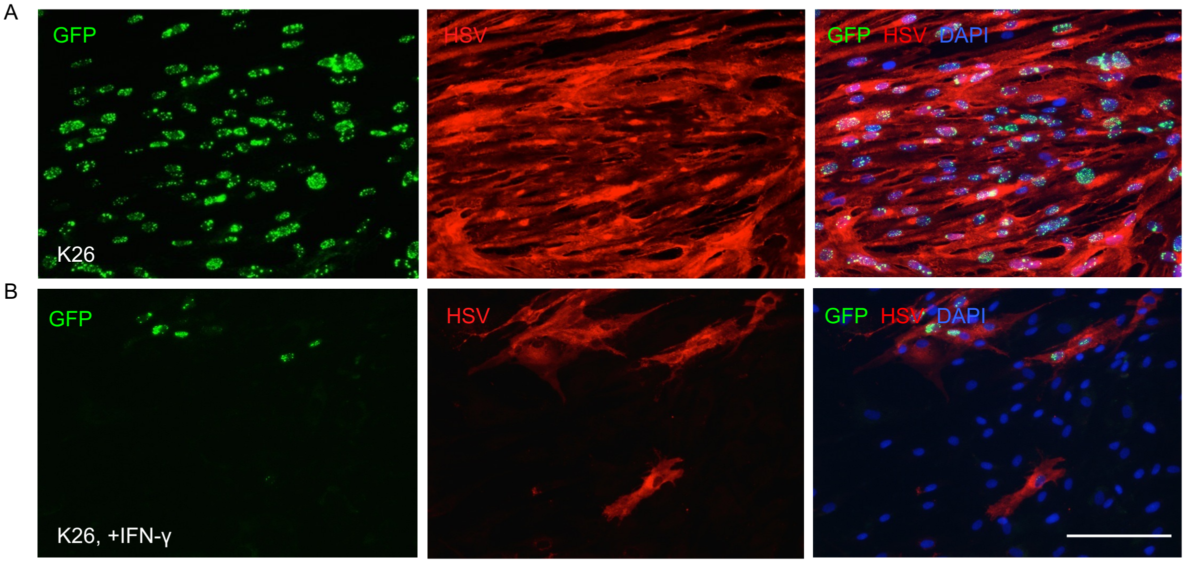

Supplementary Figure 4. **IFN-γ inhibition of HSV gene expression in primary human fibroblasts.**
GFP and HSV viral antigen expression in K26 infected MOI = 1, 16 h.p.i. human dermal fibroblasts mock-treated (**A**) or pretreated with IFN-γ 100 U/ml for 48 hours (**B**). A rabbit polyclonal antibody used to detect all HSV protein expression. Scale bar, 100 μm.

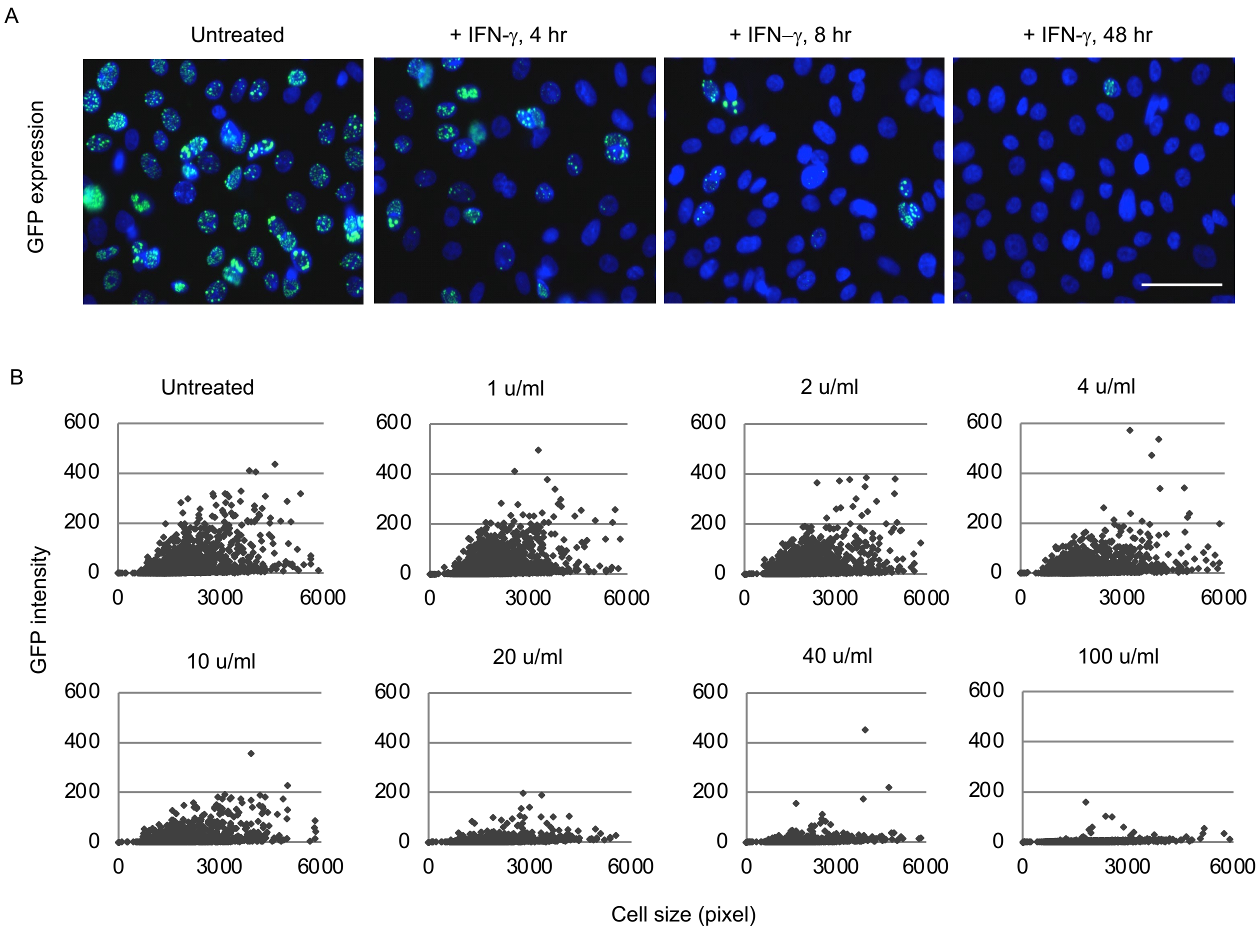

Supplementary Figure 5. Time- and dose-dependent inhibitory effect of IFN-γ on HSV infection.
(**A**) Representative raw images depicting GFP expression in K26 infected cells pretreated with IFN- γ under different times. Primary human keratinocytes, either mock-treated or pretreated with IFN-γ 100 U/ml for the duration of 4, 8 and 48 hours. Scale bar, 50 µm. MOI=1, 16 h.p.i. (B) Dot plot showing GFP expression in K26 infected human keratinocytes either mock-treated or pretreated with IFN-g at 1, 2, 4, 10, 20, 40, or 100 U/ml concentration for 48 hours. MOI=1, 16 h.p.i.

Supplementary Table 1: Significantly enriched GO terms of IFN-γ regulated genes in primary human keratinocytes

GO terms	FDR	Gene up regulated	Gene down regulated
Antigen processing and presentation of exogenous peptide antigen via MHC class II	0.008	4	0
Regulation of cell motility	0.010	14	18
Antimicrobial humoral response	0.011	3	0
Lipid transport	0.018	13	11
Negative regulation of transcription regulator activity	0.025	6	6
Antigen processing and presentation of endogenous peptide antigen via MHC class I	0.043	4	0
Inflammatory response	0.050	41	14
Innate immune response	0.050	41	5
Response to virus	0.050	35	4

Supplementary Table 2. T-test for whether individual IMCIA gene expression level higher at week 2 versus control

Signature genes	Difference, experimental minus control	Unadjusted p-value	Benjamini-Hochberg adjusted p-value	Li & Ji adjusted p-value
APOBEC3G	0.020	0.895	0.895	0.895
FOSL1	-0.047	0.864	0.889	0.888
PLSCR1	0.113	0.648	0.687	0.685
CCL8	0.154	0.611	0.669	0.666
MYD88	-0.117	0.604	0.669	0.666
DDX58	0.064	0.600	0.669	0.666
ISG15	0.198	0.410	0.495	0.491
UNC93B1	0.214	0.305	0.381	0.378
HERC5	0.083	0.212	0.275	0.272
ISG20	0.177	0.154	0.207	0.204
STAT2	0.080	0.129	0.181	0.178
IRF9	0.114	0.125	0.181	0.178
CXCL10	0.854	0.095	0.145	0.142
IFITM1	0.177	0.085	0.135	0.132
RSAD2	0.494	0.083	0.135	0.132
MX1	0.117	0.078	0.135	0.132
MICB	0.153	0.078	0.135	0.132
ZC3HAV1	0.114	0.071	0.135	0.132
IL15	0.705	0.046	0.094	0.090
EIF2AK2	0.192	0.043	0.093	0.089
IFI16	0.113	0.042	0.093	0.089
IFIH1	0.874	0.041	0.093	0.089
BCL3	0.522	0.026	0.070	0.066
BST2	0.230	0.025	0.070	0.066
MX2	0.682	0.021	0.067	0.061
CCL5	0.252	0.019	0.066	0.060
TRIM22	0.225	0.017	0.066	0.060
IFI44	0.279	0.012	0.052	0.046
IFI35	0.176	0.008	0.038	0.032
IRF7	0.274	0.007	0.038	0.032
B2M	0.124	0.006	0.038	0.032
HLA	0.122	0.004	0.038	0.030
IFITM2	0.107	0.002	0.026	0.019
STAT1	0.354	0.002	0.026	0.018
IFITM3	0.164	0.000	0.010	0.004

Supplementary Table 3. T-test for whether individual IMCIA gene expression level higher at week 8 versus control

Signature genes	Difference, experimental minus control	Unadjusted p-value	Benjamini-Hochberg adjusted p-value	Li & Ji adjusted p-value
UNC93B1	-0.030	0.877	0.877	0.877
APOBEC3G	0.028	0.756	0.778	0.778
CCL8	0.109	0.721	0.765	0.764
IL15	0.158	0.585	0.639	0.638
IRF9	0.043	0.368	0.415	0.414
MYD88	0.057	0.335	0.391	0.390
PLSCR1	0.262	0.282	0.340	0.338
IFITM1	0.214	0.264	0.330	0.328
B2M	0.043	0.232	0.301	0.299
MICB	0.187	0.189	0.255	0.253
FOSL1	0.226	0.163	0.228	0.226
ZC3HAV1	0.069	0.160	0.228	0.226
BCL3	0.251	0.141	0.215	0.212
HLA	0.029	0.129	0.205	0.203
EIF2AK2	0.444	0.118	0.196	0.193
DDX58	0.123	0.086	0.150	0.147
RSAD2	0.453	0.068	0.125	0.123
IFIH1	0.653	0.054	0.105	0.103
HERC5	0.158	0.043	0.087	0.085
IFI35	0.084	0.038	0.083	0.081
IFITM2	0.070	0.035	0.082	0.079
ISG20	0.199	0.033	0.082	0.079
STAT2	0.072	0.032	0.082	0.079
BST2	0.208	0.023	0.066	0.064
TRIM22	0.181	0.016	0.051	0.049
CCL5	0.201	0.012	0.042	0.040
IFI16	0.150	0.008	0.033	0.031
ISG15	0.504	0.007	0.032	0.030
CXCL10	0.963	0.006	0.032	0.029
IFITM3	0.131	0.005	0.029	0.027
MX2	0.700	0.004	0.025	0.022
IRF7	0.284	0.003	0.025	0.022
STAT1	0.234	0.003	0.025	0.022
MX1	0.120	0.001	0.019	0.014
IFI44	0.313	0.000	0.013	0.008
